# Major expansion in the human niche preceded out of Africa dispersal

**DOI:** 10.1038/s41586-025-09154-0

**Published:** 2025-06-18

**Authors:** Emily Y. Hallett, Michela Leonardi, Jacopo Niccolò Cerasoni, Manuel Will, Robert Beyer, Mario Krapp, Andrew W. Kandel, Andrea Manica, Eleanor M. L. Scerri

**Affiliations:** 1 Department of Anthropology, Loyola University Chicago, Chicago, IL USA; 2 Human Palaeosystems Group, Max Planck Institute of Geoanthropology, Jena, Germany; 3 Department of Zoology, University of Cambridge, Cambridge, UK; 4 Natural History Museum, London, UK; 5 Department of Biology, Loyola University Chicago, Chicago, IL USA; 6 Department of Early Prehistory and Quaternary Ecology, Eberhard Karls University of Tübingen, Tübingen, Germany; 7 Palaeo-Research Institute, University of Johannesburg, Johannesburg, South Africa; 8 The Role of Culture in Early Expansions of Humans (ROCEEH), Heidelberg Academy of Sciences and Humanities at Eberhard Karls University of Tübingen, Tübingen, Germany; 9 Department of Classics and Archaeology, University of Malta, Msida, Malta; 10 Department of Prehistoric Archaeology, University of Cologne, Cologne, Germany

**Keywords:** Archaeology, Evolutionary ecology, Ecological modelling

## Abstract

All contemporary Eurasians trace most of their ancestry to a small population that dispersed out of Africa about 50,000 years ago (ka)^1–9^. By contrast, fossil evidence attests to earlier migrations out of Africa^10–15^. These lines of evidence can only be reconciled if early dispersals made little to no genetic contribution to the later, major wave. A key question therefore concerns what factors facilitated the successful later dispersal that led to long-term settlement beyond Africa. Here we show that a notable expansion in human niche breadth within Africa precedes this later dispersal. We assembled a pan-African database of chronometrically dated archaeological sites and used species distribution models (SDMs) to quantify changes in the bioclimatic niche over the past 120,000 years. We found that the human niche began to expand substantially from 70 ka and that this expansion was driven by humans increasing their use of diverse habitat types, from forests to arid deserts. Thus, humans dispersing out of Africa after 50 ka were equipped with a distinctive ecological flexibility among hominins as they encountered climatically challenging habitats, providing a key mechanism for their adaptive success.

## Abstract (web summary)

Analysis of species distribution models in a pan-African database comprising chronometrically dated archaeological sites over the past 120,000 years shows major expansion in the human niche from 70 ka, driven by adaptation to diverse habitats.

## Main

Genetic evidence, including patterns of interbreeding with Eurasian hominins, indicates that all contemporary human populations outside Africa derive most of their ancestry from a worldwide expansion that began approximately 50 ka (refs. ^1–9^). However, the fossil record shows that earlier dispersals also occurred^10–16^. These dispersals out of Africa probably took place during repeated humid episodes within the Saharo-Arabian arid belt, most notably during the Last Interglacial, approximately 125 ka (refs. ^10,17^). An important question, therefore, concerns why these earlier dispersals were not successful enough to contribute any detectable genetic ancestry of non-African populations today, with early *Homo sapiens* failing to establish long-term viable populations in areas beyond Africa.

Researchers have proposed a range of explanations for the late dispersal of modern humans from Africa. Studies have considered that abrupt climate change in Africa^18^ and large shifts in human cognition, technology and subsistence permitted the development of new ‘niche-broadening’ innovations, such as the distinctly human ability to communicate symbolically or develop projectile weaponry^19–23^. However, complex weaponry has now been shown to have notable time depth in Africa^24,25^ and recurrent markers of symbolic behaviour are present at least during Marine Isotope Stage 5 (MIS 5, 130–71 ka) in both Africa^26–30^ and Southwest Asia^29,30^. By around 60 ka, the Middle Stone Age/Middle Palaeolithic archaeological record of Northeast Africa and neighbouring regions of Southwest Asia had become rather generic^31,32^, with no particularly notable, regionally specific shared characteristics. More broadly, the African archaeological record features complex, nonlinear trajectories of cultural change^33,34^. These often include long periods of stasis punctuated by relatively brief pulses of innovation in different African regions^35–41^ thousands of years before the advent of Eurasian founder populations^10,42^. Large, cumulative changes in the archaeological record also post-date the 60–50-ka time frame^10^, suggesting that the success of modern human founding populations in Eurasia did not rest on a particular widespread technological innovation or sudden increased cognitive capacity^43^. More recent studies have focused on a variety of interrelated explanations rooted in demography. In particular, the apparent geographic expansion of Middle Stone Age archaeological sites in Africa 125–50 ka has been considered to reflect a possible growth in population coupled with an expansion inside Africa of an ancestry similar to the major expansion wave^1,44–46^. This population expansion is also thought to include greater population density^47^ and increases in connectivity through long-distance social networks^48^, all of which may have acted as ‘push’ factors. Building on quantitative studies of niche changes associated with hominin species^49^, and with particular Middle Stone Age lithic industries^22^, the spatial patterning of sites has also been used to suggest a uniquely human adaptation to a ‘generalist–specialist’ niche^50^. However, none of these hypotheses have been quantitatively assessed on a continental scale and few have been examined in terms of the predictions of suitable theoretical models, risking a proliferation of post-hoc explanations^51,52^. Because human expansion within and out of Africa can be linked to expansions in niche breadth under an ideal free distribution^53^ model, we explored successful out of Africa in terms of expansions of the human niche. As population densities increase, human settlement in more challenging environments becomes profitable. Under such conditions, in which the critical population mass required for successful out of Africa movements is reached and the ecological resilience of humans increases, expansions can occur^54,55^.

Here we specifically test whether the niche of Pleistocene *H. sapiens* expanded or contracted within Africa before major dispersals out of the continent. We define niche as the ensemble of bioclimatic factors determining where the species can survive and reproduce^56^. To answer this question, we build on previous studies^22,57–60^ and use a SDM approach^61^ to measure changes in the breadth of the Pleistocene human niche within Africa. Our results, tested against two different sets of palaeoenvironmental simulations^62–64^, formally demonstrate that human niche expansion precedes and coincides with successful later dispersal out of Africa.

## Testing for the presence of niche change

**Fig. 1:**
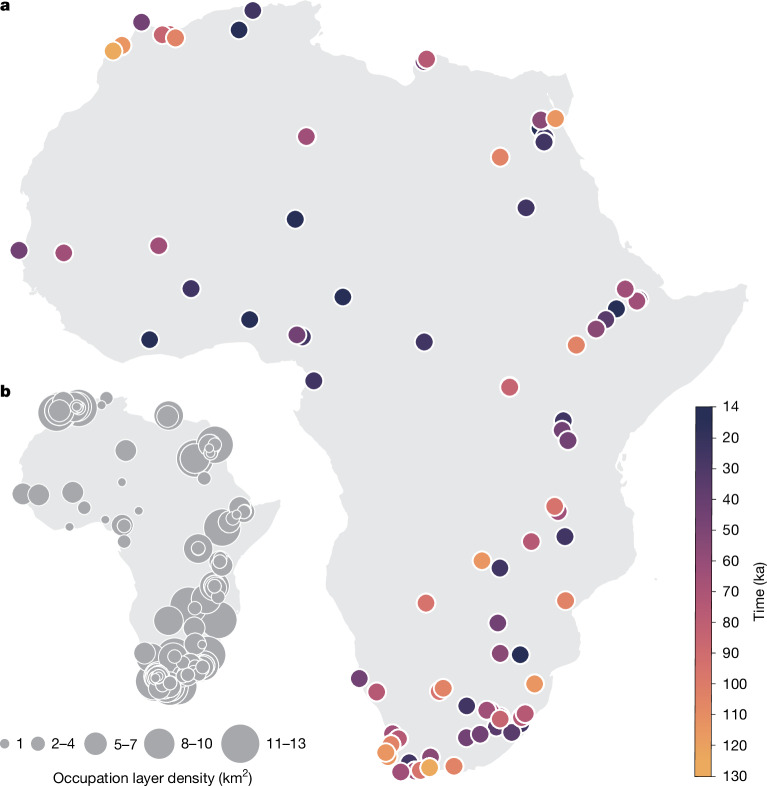
Map of dated archaeological sites in Africa. **a**, Location and time ranges of archaeological sites. **b**, Density of radiometrically dated archaeological layers per square kilometre used in this study (see Supplementary Table 1). The sample size is 479.

To reconstruct the human niche in Africa over time, we took a pan-African approach and assembled a comprehensive and curated database of radiometrically dated Pleistocene occupation layers from archaeological sites in the time frame 120–14 ka, excluding any site with an age uncertainty greater than 20,000 years (see Methods for details on the choice of sites and Supplementary Table 1 for sites included here). For each retained occupation layer (Fig. 1), which represents a confirmed presence, we then extracted reconstructed palaeoclimatic variables and biomes^62^ (based on vegetation simulations with the BIOME4 model) associated with the location and date for a total of 479 radiometrically dated occurrences. We focused on five bioclimatic and vegetation variables that seemed to be the most informative to reconstruct habitat suitability based on our data (see Methods): (1) leaf area index (LAI); (2) temperature annual range (BIO 7); (3) mean temperature of the wettest quarter (BIO 8); (4) mean temperature of the warmest quarter (BIO 10); and (5) precipitation of the wettest quarter (BIO 16). Because of the limited amount of data for each specific biome, the vegetation simulations from BIOME4 were combined into three main classes: forest, savannah and desert (see Methods and Supplementary Table 4). We used two sets of palaeoclimatic simulations based on: (1) the Hadley Centre Coupled Model, version 3 (HadCM3 (ref. ^62^)), with results presented in the main text, and (2) the Transient Community Earth System Model (PCESM^63,64^), with results shown in Extended Data Fig. 8a. Although the two sets of simulations have a high degree of correlation, the magnitude in fluctuations for both temperature and precipitation differs through time and space (Extended Data Fig. 8a); despite these discrepancies, all of the key results are qualitatively robust to the palaeoclimate model used.

To reconstruct the human niche over time, we used a SDM approach fitted to the five bioclimatic variables. Biome classes were not used as inputs for the SDMs but only to characterize the environment of cells predicted as habitable by the models. Conventionally, niche changes are tested by fitting SDMs to different time periods and comparing the inferred niches. However, data for Pleistocene humans are very sparse and it would not be possible to create several time slices with enough occurrences. Instead, we use generalized additive models (GAMs) to fit the full time series as a single dataset^65^. With this approach, it is possible to formally test for changes in the niche over time by comparing a GAM in which the effect of each environmental variable in predicting occurrences is constant through time (fixed-niche GAM) against a GAM in which the effect is allowed to vary (changing-niche GAM; formally, by fitting an interaction between each predictor and time; see Methods).

**Fig. 2:**
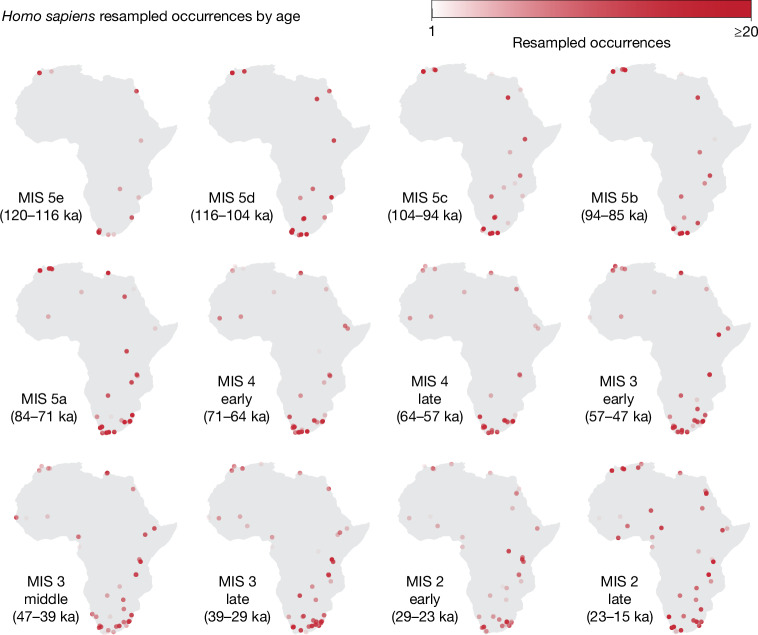
*Homo sapiens* resampled occurrences by MIS. To handle chronological uncertainty of archaeological layers, we resampled dates from the appropriate ranges: the saturation of the points indicates the number of resamples that were attributed to a given location for each MIS (darker points come from layers with low uncertainty for which most dates fall within the same MIS; lighter points represent layers with high uncertainty for which resamples are spread among several MISs). Details of the resampling approach and chronological subdivisions within MIS 4, MIS 3 and MIS 2 are described in Methods.

**Extended Data Fig. 1:**
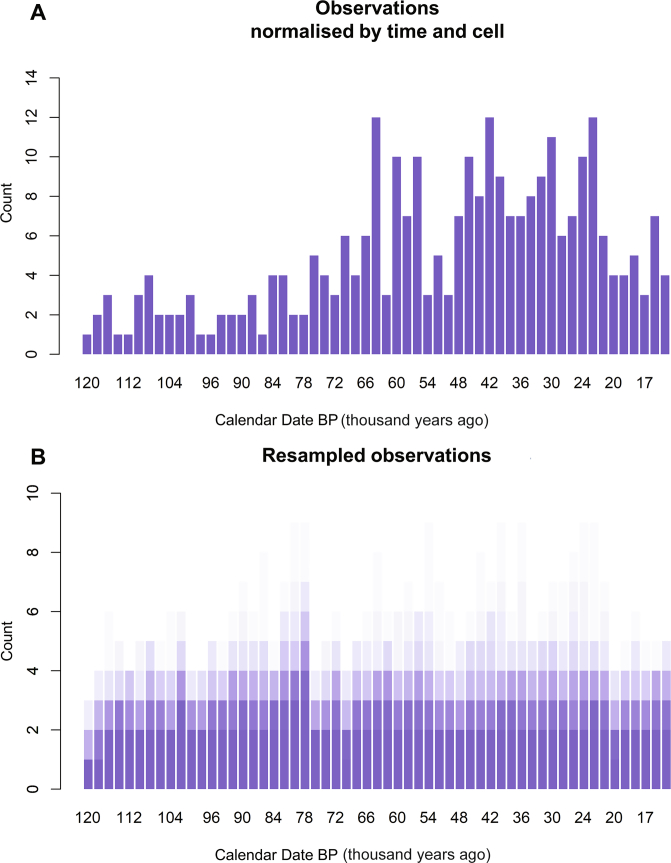
Histogram of archaeological occurrences and resampled occurrences through time. **a**, Histogram of archaeological occurrences (in ka), using the mean age of each layer. **b**, Histogram of resampled occurrences through time (in ka) (100 resamples per layer).

The number of dated archaeological layers in our dataset varies with time, with an increase in abundance towards more recent periods (Extended Data Fig. 1a). This pattern probably results from increased chances of preservation in the more recent past, thus constituting a form of sampling bias that could distort the results. To avoid such a bias, we randomly subsampled the observations to generate a homogeneous distribution in the number of occurrences through time (Extended Data Fig. 1b). We repeated this process 100 times to generate several ‘standard-effort datasets’ to explore the stochastic effect of such subsampling (Fig. 2). We took into account the chronological uncertainty associated with each radiometric date as follows: in each of these datasets, we associated each occurrence with a date sampled from its whole available chronological range (following a normal distribution around the mean). Finally, for each standard-effort dataset, we then quantified the background distribution of the climate variables against the occurrences, sampling 200 random locations matched in time for each occurrence. Next, we fitted a fixed-niche GAM and a changing-niche GAM to each set of standard-effort occurrences coupled with the associated pseudo-absence points, giving us 100 repeats of our analysis. Formal model comparisons using the Akaike information criterion (AIC) strongly supported the changing-niche GAM in 91 of 100 repeats (see Methods). To understand the changes in the niche detected by the model, we combined repeats into an ensemble^66^; specifically, we combined repeats with the AIC supporting a changing-niche GAM and a Boyce continuous index (BCI; a measure of goodness of fit for presence-only data)^67^ larger than 0.7 and investigated changes over time. We also created an ensemble from the repeats with AIC < 2 (hence not substantially supporting a changing-niche model) using the fixed-niche GAMs, which provides us with a null model of the changes owing to climatic fluctuations over time in the absence of any niche change. A qualitatively similar pattern can be observed on the same analyses performed using the PCESM palaeoclimatic simulations^63^.

## Geographic range expanded 70 ka

**Fig. 3:**
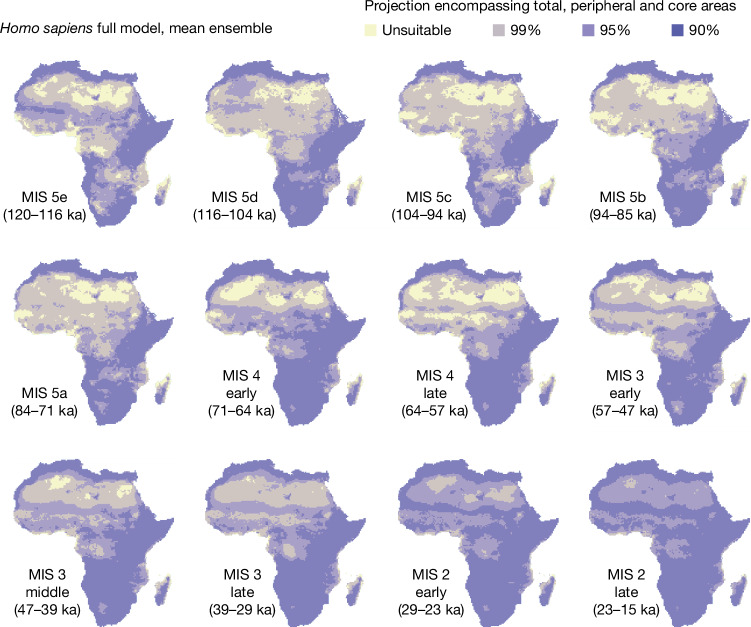
Projection of habitat suitability through time for the changing-niche model. Mean ensemble; see Methods. Yellow represents unsuitable areas and the purple colours represent increased suitability from lighter to darker shades. These are calculated as total, peripheral and core areas, encompassing 99%, 95% and 90% of archaeological occurrences, respectively. The maps for each MIS are produced by calculating the mean of the habitat suitability for each individual cell within the time slices of interest. Chronological subdivisions of MIS 4, MIS 3 and MIS 2 are described in the Methods. The climate used is that of Beyer et al.^62^ with added Heinrich events. See Extended Data Fig. 8b for equivalent results using PCESM^63,64^.

**Extended Data Fig. 2:**
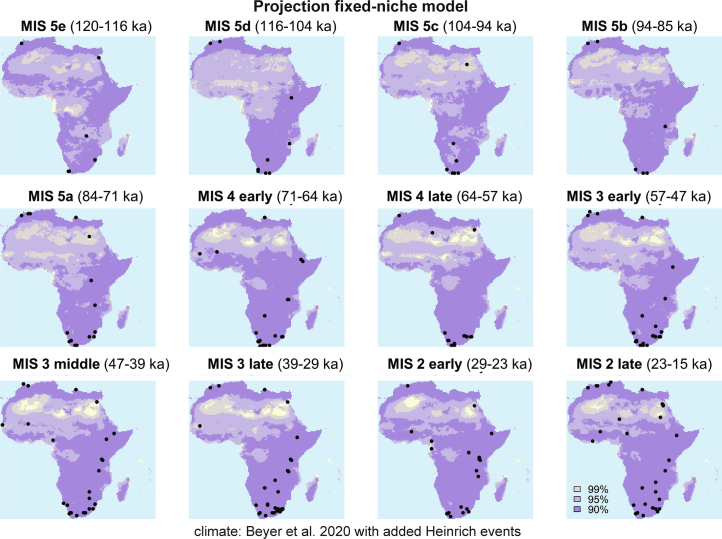
Projection of the fixed-niche model through time (using climate reconstructions from Beyer et al.^57^ with added Heinrich events). The core (90% of occurrences), peripheral (95% of occurrences) and total area (99% occurrences) are shown.

The ensemble of changing-niche GAMs showed a substantial increase in the habitable geographic range of Pleistocene humans within Africa over time (Fig. 3). The most notable increase was in West, Central and North Africa beginning roughly 70 ka at the onset of MIS 4. This large increase in geographic range around 70 ka was followed by a range expansion encompassing most of Africa beginning about 29 ka, during MIS 2 (Fig. 3). These changes were the result of niche expansion, as the fixed-niche GAM predicted a relatively stable and extensive inhabitation of the whole continent (Extended Data Fig. 2). Qualitatively similar results were obtained performing the same analyses with the PCESM climate simulations (Extended Data Figs. 8b and 10).

## Changes in niche breadth

**Fig. 4:**
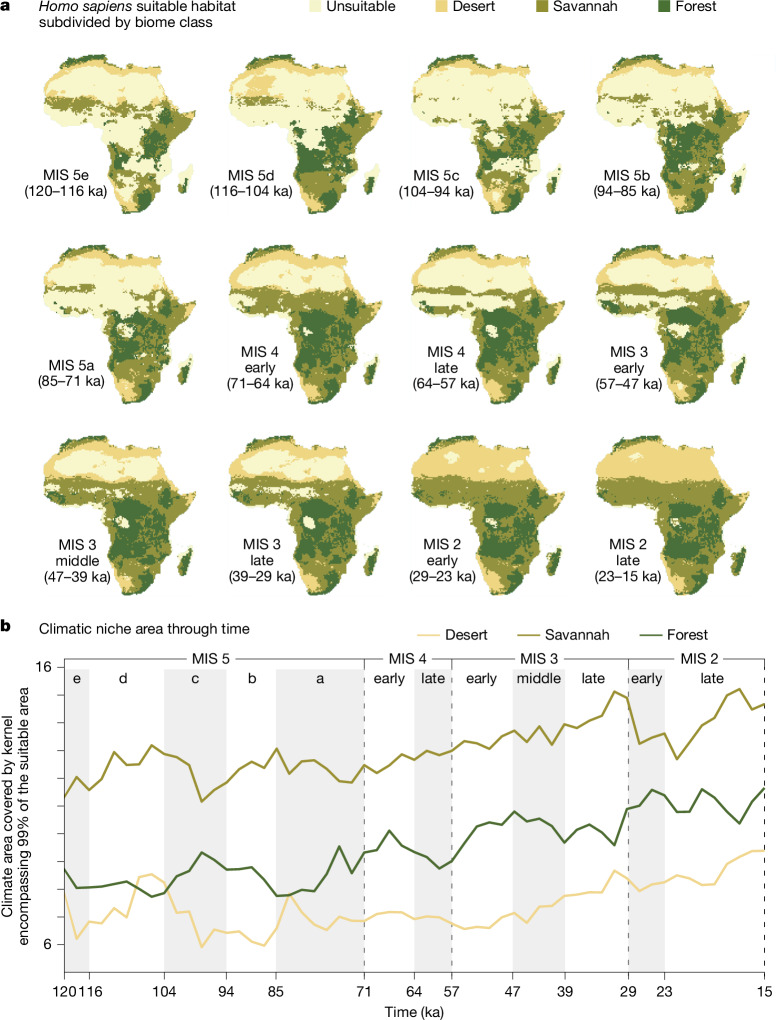
*Homo sapiens* suitable habitat and climatic niche area through time. **a,** *Homo sapiens* suitable habitat (combined core and peripheral areas) subdivided by biome class (desert, savannah and forest). **b**, Climatic niche area through time. The niche area is calculated on the plot of the first two principal components of the climatic variables by estimating the 2D 99% kernel encompassing the suitable cells (combined core and peripheral areas) for each biome class. Equivalent results using PCESM^63,64^ are shown in Extended Data Fig. 4a.

**Extended Data Fig. 3:**
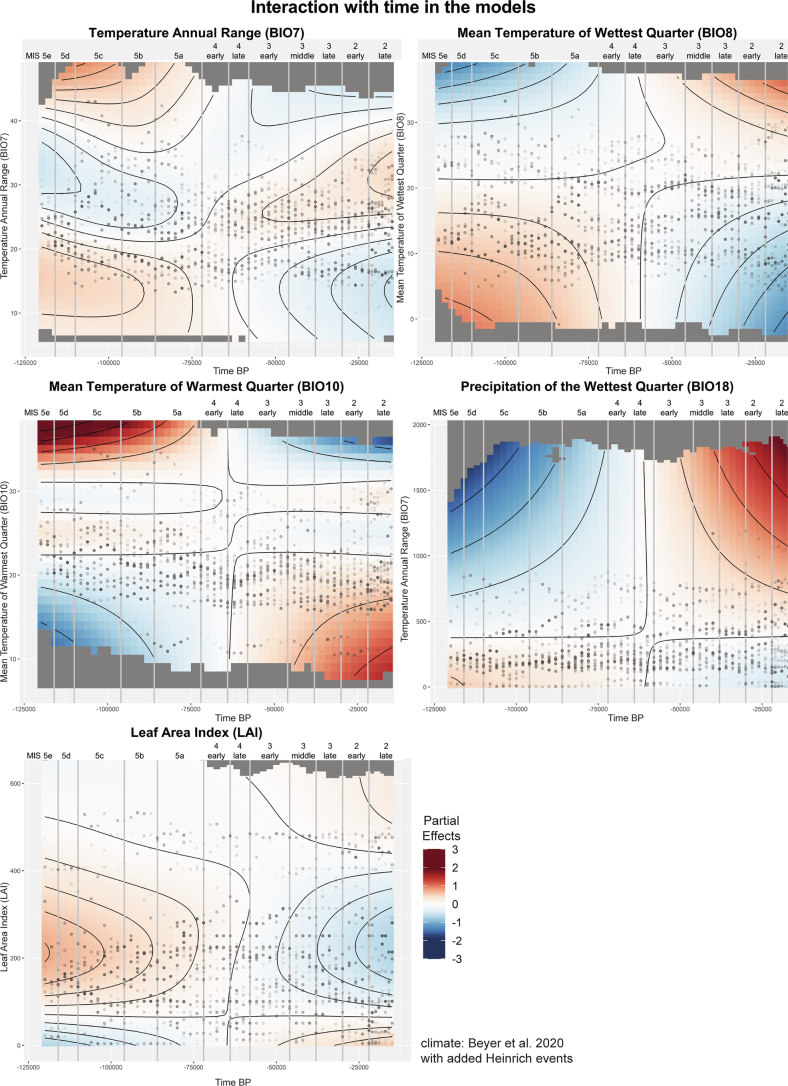
Interaction with time in the models built using the Beyer et al.^57^ climatic reconstructions with added Heinrich events. *Homo sapiens* resampled occurrences are shown as grey dots (overlapping points are shown in darker shades based on their number). The partial effects of the GAMs (*z* axis, colour shading) show the interaction between the variable of interest (*y* axis) and time (*x* axis). Here we present the mean values of the partial effects plots over 100 repeats. The red shading represents favourable variable space (used more than expected by chance given the availability in the whole continent) and blue represents less favourable variable space. The *y* variables used in the GAMs are: temperature annual range (BIO 7), mean temperature of the wettest quarter (BIO 8), mean temperature of the warmest quarter (BIO 10), precipitation of the wettest quarter (BIO 16) and LAI.

**Extended Data Fig. 4:**
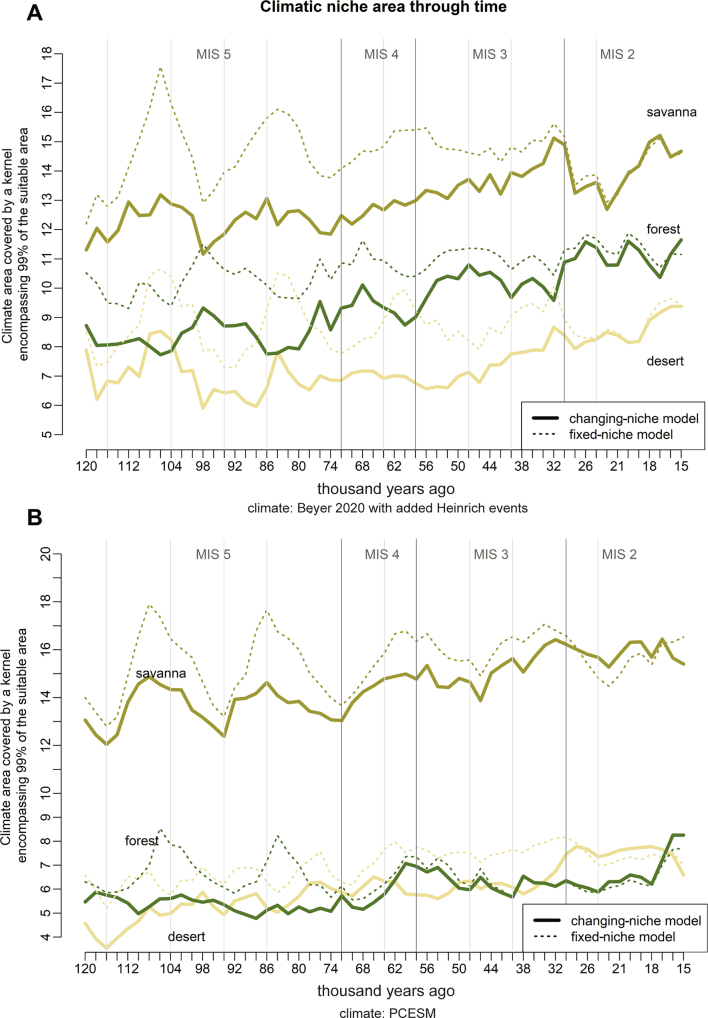
Comparison of the niche area. Encompassing 95% of presences covered through time for each of the main biomes in the changing-niche model (solid lines) and the fixed-niche model (dashed lines). **a**, Beyer et al.^57^ modified to include Heinrich events. **b**, PCESM^58,59^.

To better understand the relationship between these changes in geographic range and the realized niche breadth as defined by the ensemble of changing-niche GAMs (Fig. 4a), we investigated the changes in niche breadth for each biome class under both the changing-niche and the fixed-niche models. We created a principal component analysis of the environmental variables used in the GAMs, pooling over all time steps, to generate a synthetic 2D space that describes climatic variability over the period of interest. For each time step, we then measured the area of a kernel fitted over the locations that were predicted as suitable by the respective model in each biome class (Fig. 4b). We also explored the interaction plots (that is, the partial effects of the interaction between each bioclimatic variable and time) of the ensemble of changing-niche GAMs (Extended Data Fig. 3) to understand the exact changes in the niche, as these represent the (theoretical) full niche and are not affected by the availability of different climatic conditions. Figure 4b shows the size of the realized niche for each biome within Africa, allowing the relationship between humans and climate to change (changing-niche model), whereas Extended Data Fig. 4 compares (for both palaeoclimatic simulations used) this pattern with the plot assuming this relationship to be constant (fixed-niche model). As a result, the latter represents how human distribution would have changed only following climatic fluctuations.

As shown in Fig. 4b, after fluctuations around a relatively stable level up to MIS 5a, the potential use of savannah and forest started to increase steadily between MIS 5a and MIS 4. The increase was not linked to an increase in availability of those habitats, as it was not reproduced by the fixed-niche GAM (see Extended Data Fig. 4a for an equivalent plot to Fig. 4b). Rather, use of areas in which the wettest quarter was both warmer (BIO 8) and had greater precipitation (BIO 16; see Extended Data Fig. 3) increased, thus expanding the niche progressively into the forests. The use of deserts was more variable, in part because of fluctuations in the degree of aridity of deserts available. During wetter periods, we found an increase in the niche space of deserts that were suitable for human habitation, as they were less extreme in their climatic characteristics. We observe this effect in the reconstructed realized niche area of the fixed-niche GAM, in which changes through time are only linked to climatic changes affecting available climate conditions (see Extended Data Fig. 4a). The key difference between the changing-niche and fixed-niche GAMs is that, in the former, after a peak during early MIS 5a owing to more favourable climate, deserts remain in greater use than during previous, less favourable dips. This stable expansion into the desert is then maintained until MIS 3, when we see another progressive increase in the use of desert habitats. This was not seen in the fixed-niche GAM. Mechanistically, the increased use of deserts was underpinned by an increased use of regions with larger annual temperature ranges (BIO 7 in Extended Data Fig. 3) and a progressive increase in the ability to survive in areas with low LAI (that is, with sparse vegetation; LAI in Extended Data Fig. 3). Although slightly muted, the same patterns were observed when repeating the analyses using the biome classes based on PCESM^64^ (Extended Data Figs. 4b and 9).

## Discussion

Our results show that the human niche progressively expanded to include more habitat types beginning around 70 ka and that this expansion peaked at about 50 ka, coinciding with successful dispersals out of Africa. A second expansion was observed starting approximately 29 ka; by MIS 2, humans occupied all African regions and ecosystems, with terminal Pleistocene societies engaging with a range of new behaviours, including semi-sedentism^68^, evidence of persistent macroscale social networking^69^ and increased territoriality and interpersonal violence^70^. Our findings are consistent using two different and independently developed sets of palaeoenvironmental simulations. The expansion of the human niche beginning around 70 ka in Africa is driven by a gradual increase in human preference for forest and desert biomes, allowing them to expand into regions that were previously rarely populated: (1) forests of West Africa, (2) forests of Central Africa and, eventually, (3) arid Saharan regions and semi-arid Sahelian regions of North Africa. This increased ability to adapt to new habitats, ranging from the extremes of equatorial forests to arid deserts, would have allowed these populations of humans the ecological flexibility to tackle a range of new environmental conditions encountered during the expansion out of Africa, allowing them to succeed where earlier migrations out of Africa had previously faltered. The expansion of the human niche in Africa starting around 70 ka, therefore, offers an explanation for the successful worldwide expansion of human populations about 50 ka and is consistent with predictions informed by the ideal free distribution about the consequences of population expansions.

These results also offer insights into changes in the archaeological record between approximately 70 and 50 ka, a time when diverse regions feature distinctive cultural trajectories (see Supplementary Information). In particular, ecosystem engineering through widespread burning^71^, water storage technology^72,73^ and expanded diet breadth^74^ have been previously documented in Africa broadly within this time frame and slightly before. These findings may reflect behavioural preconditions and manifestations of the expansion of the human niche into new habitats. It is important to stress that our pan-African results do not imply a unique, unified process over the whole continent. It is indeed more likely that it is linked to a few specific cultural complexes. More research must be carried out to unravel what drove the changes that we observe.

We emphasize that, although we document a niche expansion, this does not necessarily reflect an increase in overall population size and it may not be possible to distinguish between the effects of population size increase versus increases in density within reduced niche space^47^. For example, the new human niche in arid regions was unlikely to have sustained large or stable population sizes owing to low carrying capacity^75^. Instead, the expansion of the human niche indicates that humans were able to move among several habitats and locations, probably increasing the encounter rate between different groups over time. If this were the case, it may explain why the constellation of features that define humans today became fixed in individuals between 100 and 50 ka (ref. ^42^). It would also provide independent support for the hypothesis that there could have been an expansion inside Africa of an ancestry that is similar to the one that expanded into Eurasia after about 60 ka (refs. ^44,45^).

The period roughly 70–50 ka is climatically complex. Although the Heinrich Event 6 brings an overall cooler and drier spell owing to the weakening of the Atlantic Meridional Overturning Circulation^76^, the magnitude of this effect differs across regions of Africa. Notably, the proxy record points to repeated shifts between dry and wet spells^77^, with suggestions of a broader humid period that might have affected a large part of the continent following Heinrich Event 6 (ref. ^78^). Our runs of HadCM3 (combining Beyer et al.^62^ and Armstrong et al.^79^) capture this temporal and spatial heterogeneity (Extended Data Fig. 8a); in PCESM^63,64^, which does not explicitly include Heinrich Event 6, the pattern is more muted (Extended Data Fig. 8a). The impact of Heinrich Event 6 for human evolution has mostly been framed in terms of its role in dispersals out of Africa. It has been argued that the aridification of Northern and Eastern Africa might have acted as a push event favouring migrations of humans out of Africa^80^; however, it should also be noted that, because of the cooling effect and its heterogeneous impact, this period potentially provided corridors that might have favoured movement out of the continent^81^. A similar argument can be made for the onset of MIS 2 around 29 ka, when a period of heterogeneous aridification prompted a further move into more arid habitats. Given our results, it is plausible that the instability of climate in these periods might have favoured the evolution of a broader niche in humans, further enhancing their ability to cope with the diverse habitats that distinguish the genus *Homo*^64^.

By modelling the human niche within Africa from 120 to 14 ka, we show that successful expansion into Eurasia and the long-term establishment of populations there were part of a process that fundamentally started within Africa. Essentially, we document the inception of an unquestionably African process originating 70 ka that has resulted in today’s unparalleled human ecological plasticity. Our results, therefore, provide a new basis for understanding and investigating our species’ global dispersal.

## Methods

### African Pleistocene archaeological deposits included in models (script 1)

Coordinates of archaeological sites are essential for SDMs, as these models are built on the accurate input of spatial occurrence/presence data. Chronometrically dated archaeological layers are also necessary for linking occurrences to the appropriate local climate simulations used in SDMs. Radiometric dating methods provide age estimations before present that are measured on the basis of physical and chemical changes that occur over time^82^. Radiocarbon (^14^C), uranium–thorium (U-Th or U-series), optically stimulated luminescence, thermoluminescence and electron spin resonance—among others—are dating methods that provide age estimates in calendar years before present^83^. In cases in which the age of an archaeological layer was estimated only by non-radiometric methods, it was excluded from subsequent analyses. Non-radiometric dating methods include biochronology, typology and palaeomagnetism. If an archaeological layer was not dated, it was likewise excluded from subsequent analyses. Following the removal of archaeological layers that were not subjected to radiometric dating techniques, further limits were placed on archaeological occurrences included in distribution models and niche area calculations. Any occurrence was excluded if the minimum age subtracted from the maximum age was greater than 20,000 years. These efforts resulted in a database (Supplementary Table 1) with archaeological layers that have: (1) published coordinates; (2) radiometric dates; (3) an age error range less than or equal to 20,000 years; and (4) mean age ≤120,000 and ≥14,000 years. Archaeological sites and layers were compiled from peer-reviewed journals and books published before 5 May 2021. The Role of Culture in Early Expansions of Humans (ROCEEH) has been integrating archaeological, palaeoanthropological, palaeontological and palaeobotanical information into the ROCEEH Out of Africa Database (ROAD) since 2009 (ref. ^84^). As of May 2021, the team has compiled information on more than 2,000 localities and more than 12,000 assemblages in Africa and Eurasia dated between three million and 20,000 years before present. The information in ROAD is structured into localities (sites) with dated layers that contain assemblages of finds, including artefacts, human fossils, palaeofauna and plant remains, and bibliographic sources. For this study, A.W.K. and M.W. queried ROAD to create a list of archaeological localities in Africa dating between about 500,000 and 10,000 years and reviewed the output for accuracy. The output also included the ages of the layers, as well as radiometric dating results.

### Calibration of ^14^C radiocarbon ages (script 2)

**Extended Data Fig. 5:**
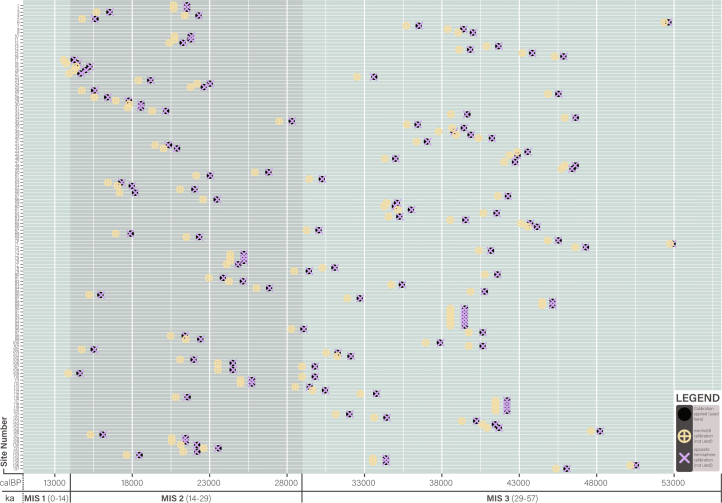
Calibration plots. The differences between the calibration applied in the present study (black), opposite hemisphere calibration that was not applied (purple) and marine calibration (orange) that was not applied.

In our database of archaeological sites, all radiocarbon dates were entered as uncalibrated ages, with the exception of Haua Fteah, for which modelled and calibrated ^14^C radiocarbon and optically stimulated luminescence/electron spin resonance ages were entered from Douka et al.^85^. If a single archaeological layer was dated with both ^14^C and further chronometric dating methods, the uncalibrated radiocarbon ages were entered in a separate column from the extra radiometric dating methods. For all uncalibrated ^14^C ages entered in our database, a basic IntCal20 (ref. ^86^) calibration for Northern Hemisphere archaeological localities and a basic SHCal20 calibration for Southern Hemisphere archaeological localities was completed using the rcarbon R package^87^. The calibrated ^14^C ages were then used to determine the mean age and age error ranges were calculated to the level of 1*σ*. If an archaeological layer was dated with ^14^C and further methods, our calibrated ^14^C mean age and 1*σ* error were then combined with the mean age and error range of the other methods to find the mean age used in our models. Note that we did not calibrate ^14^C ages using marine calibration curves (Marine20 (ref. ^88^)), nor did we calculate Δ*R* values for marine carbonates (but see Reimer and Reimer^89^ for calculation of Δ*R* for paired marine and terrestrial dates). Marine calibration curves and Δ*R* values were not included in our ^14^C calibration for two reasons: (1) for many ^14^C ages, it is unknown whether dated samples are marine or terrestrial shell and (2) Δ*R* values are determined regionally based on the nearest samples in the Δ*R* database but, for much of Africa, the sampling coverage is low. We also did not subtract 180 years before calibration from ^14^C ages obtained from ostrich eggshell^90^ (as 180 years is too short a time span to change our models that run in 1,000-year or 2,000-year intervals), nor did we set the northernmost limit for Southern Hemisphere calibration at the Intertropical Convergence Zone, as this boundary is recommended only when seasonal variations are known^91^. Instead, we set the boundary between Northern Hemisphere and Southern Hemisphere calibration at the equator, as seasonal variations are unknown for most of the archaeological sites included in the present study. Extended Data Fig. 5 shows the differences between the calibration applied in the present study, opposite hemisphere calibration that was not applied and marine calibration that was not applied. In all cases, the possible dates based on different approaches to calibration fall within a relatively narrow range that would lead, at most, to moving a site to the adjacent 2-ka time window and would have a minimal effect on the local climate assigned to that occurrence. Supplementary Table 2 shows the number assigned to each archaeological site name and layer for Extended Data Fig. 5, as well as uncalibrated ages and errors, calibrated ages and 1*σ* errors that were used in the present study and calibrated ages and 1*σ* errors that were not used in the present study.

### Subdividing MISs

To better visualize how the patterns change through time, and better compare different periods, in our figures, we subdivided MIS stages 4, 3 and 2 to create roughly equal time periods. We subdivided MIS stages 5, 4, 3 and 2 following *δ*^18^O peaks and lows from Cohen and Gibbard^92^. We subdivided MIS 5 into MIS 5e (120–116 ka), MIS 5d (116–104 ka), MIS 5c (104–94 ka), MIS 5b (94–85 ka) and MIS 5a (84–71 ka); MIS 4 was subdivided into early (71–64 ka) and late (64–57 ka); MIS 3 was subdivided into early (57–47 ka), middle (47–39 ka) and late (39–29 ka); and MIS 2 was subdivided into early (29–23 ka) and late (23–15 ka).

### Water hosing and Heinrich events

The palaeoclimatic reconstructions from Beyer et al.^62^ do not include water hosing to simulate Heinrich events. To include them, we considered simulations from Armstrong et al.^79^ (although the reference cited focuses on the Northern Hemisphere, the associated simulations of the global circulation model are available on BRIDGE^93^). In those simulations, Dansgaard–Oeschger and Heinrich events are imposed as a spatial fingerprint with time-varying amplitude to match temperature reconstruction from Greenland ice cores. The spatial fingerprint itself was derived from a freshwater hosing simulation during the Last Glacial Maximum using HadCM3B-M2.1 (ref. ^79^). As the two runs use slightly different boundary conditions, we computed anomalies for monthly temperature and precipitation between the time slices of interest (that is, with Heinrich events simulated by water hosing) and the simulations for the present, and then added those anomalies to the simulations from the present used by Beyer et al.^62^, working at the original coarse resolution of HadCM3. We then bias-corrected and downscaled those time slices using the same approach as Beyer et al.^62^, thus creating a coherent dataset, and recomputed BIOCLIM variables and ran the BIOME4 global vegetation model, modified to include water hosing and Heinrich events. At the end, we had 17 BIOCLIM variables available (BIO 2 and BIO 3 could not be computed as they are based on daily rather than monthly summaries) and two vegetation variables (net primary productivity and LAI).

### SDM methods (scripts s01 to s16)

#### Variable selection

**Extended Data Fig. 6:**
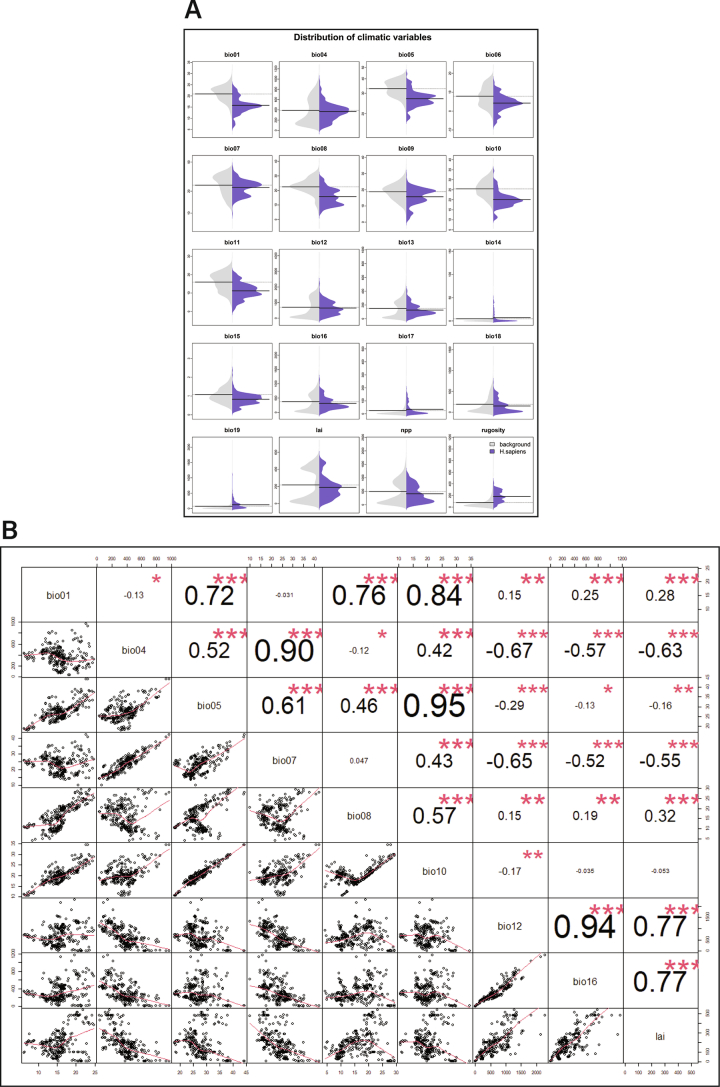
Distribution and cross-correlation of climatic variables. **a**, Distribution of climatic variables in the archaeological occurrences through time versus the whole continent (background). Climate: Beyer et al.^57^ with added Heinrich events. **b**, Cross-correlation of climatic variables (dataset: Beyer et al.^57^ with added Heinrich events) for *H. sapiens* occurrences. The lower-left triangle shows the scatter plot between pairs of variables. The upper-right triangle contains the numerical values of the cross-correlations, with bigger sizes corresponding to larger distance from 0. The asterisks in each cell represent the level of significance of the corresponding value.

Variable selection was performed using the R package tidysdm^94^. The palaeoclimatic reconstructions (Beyer et al.^62^ with added Heinrich events; see section above) were stored as a netCDF file and were accessed using the R package pastclim^95^ (dataset = “custom”). We defined which variables to use for our analyses using the equivalent of the plot_pres_vs_bg() and dist_pres_vs_bg() functions in tidysdm^94^. We used an older version of the function tidysdm::plot_pres_vs_bg() to create a violin plot comparing the distribution over the variable space of the occurrences versus a representative sample of the background. The latter was obtained by randomly drawing 10,000 points from each time slice over the whole area and period considered (Extended Data Fig. 6a). The variables for which humans seemed to occupy the climatic space differently from what was available in the environment suggest non-random use of the variable itself, supporting the idea that they would be more meaningful to reconstruct the distribution of the species (see, for reference, the function tidysdm::dist_pres_vs_bg()). Finally, among these informative variables, we extracted a set of variables that showed a correlation among each other below a threshold of 0.8 (Extended Data Fig. 6b), leaving: LAI, temperature annual range (BIO 7), mean temperature of the wettest quarter (BIO 8), mean temperature of the warmest quarter (BIO 10) and precipitation of the wettest quarter (BIO 16).

#### Chronological uncertainty and temporal sampling bias

Each radiometric date is associated with a specific chronological uncertainty, expressed as a range (most likely date and plus/minus). To take into account such uncertainty, we performed our analyses as follows. We created 100 independent datasets: for each of them, the spatial coordinates of all occurrences (= radiometrically dated archaeological layer) were associated to an age that was resampled from the whole chronological range of the date. Such sampling was performed following a truncated normal distribution identified by the mean and the plus/minus as 2*σ* (ref. ^96^).

When looking at the number of our occurrences through time (as exemplified by their mean age shown in Extended Data Fig. 1a), there is a marked increase in the number of layers observed through time. This pattern is most likely the result of a combination of an increasing number of human occupations through time, a conservation bias (with more recent traces of occupation being more likely to have survived than older ones) and the differential use of the available radiometric dating techniques (for example, ^14^C is only available up to around 50 ka). To account for all of these factors, each of the resampled datasets was randomly subsampled to generate a homogeneous distribution in the number of occurrences available through time (thus giving us constant sampling effort through time) using the following procedure. We identified that, at 78 ka, there is a boundary between an older period with a low number of occurrences (on average between zero and eight per time slice, varying on the basis of the chronological resampling) and a more recent period in which there is much more data available. For each resampling, we then subset each time slice from the more recent period to the number of occurrences observed in a randomly sampled older time slice (Extended Data Fig. 1).

To adequately represent the existing climatic space (that is, background) in our SDMs, each of these resulting, constant-effort datasets was coupled with a random sampling, for each observation, of 200 random locations matched by time. This resulted in *n* = 100 datasets (= ‘repeats’) of randomly sampled and dated occurrences and differently sampled background points. We independently repeated our analyses for each of these datasets, thus accounting for the stochasticity of resolving the time uncertainty, the sampling biases and the choice of background points. From this stage, all analyses were repeated independently using two sets of palaeoclimatic simulations: Beyer et al.^62^ with Heinrich events and PCESM^63,64^.

#### Modelling

We then used GAMs (mgcv package in R (ref. ^97^)) to fit two possible models: a ‘changing-niche’ model that included interactions of each environmental variable with time (fitted as tensor products) and a ‘fixed-niche’ model that included the environmental variables as covariates but lacking interactions with time^65^. Thus, for the fixed-niche model, the mgcv GAM formula is

gam(obs~ti(bio07, k=4) + ti(bio08, k=4) + ti(bio10, k=4) +

      ti(bio16, k=4) + ti(lai, k=4),

     data=data, family='binomial')

Here human presence/absence (obs) is modelled as the sum of five nonlinear functions (of the four bioclimatic variables and LAI). Each function ti() denotes a tensor product smooth term created using thin plate regression splines, in which each spline is composed of k=4 basis functions. family='binomial' indicates that the response variable (obs) follows a binomial distribution and the link function is the logit function. data denotes the R data frame containing the response and predictor variables.

For the changing-niche model, we have

gam(obs~ti(bio07, k=4) + ti(bio08, k=4) + ti(bio10, k=4) +

      ti(bio16, k=4) + ti(lai, k=4) + ti(time_bp, k=4) +

      ti(time_bp, bio07, k=4) + ti(time_bp, bio08, k=4) +

      ti(time_bp, bio10, k=4)+ ti(time_bp, bio16, k=4) +

      ti(time_bp, lai, k=4),

       data=data, family='binomial')

This model uses an extra five bivariate functions, each of which includes time before present (time_bp) as a second argument, as well as the relevant bioclimatic variable or LAI. Note that both models use bioclimatic and LAI values corresponding to the time and location of the human presence data (obs), but only the second model considers nonlinear interactions between the bioclimatic and LAI variables with time, whereas the first model only includes a univariate time function term. For a formal definition of the univariate and bivariate thin place regression splines, ti(x) and ti(t,x), respectively, we refer readers to Wood^98^ (specifically section 2, starting on page 1,026, as well as subsection 2.1 ‘Nesting and ANOVA decomposition’ on page 1,028, which provides a rationale for how the models are compared). A comprehensive description of how GAMs are fitted in mgcv is given by Wood^97^.

For all GAMs, we set *k* = 4 as the maximum threshold for the degrees of freedom of the splines; this value provides a reasonable compromise between allowing the relationship to change through time but avoiding excessive overfitting^99^.

#### Residual checks

For each repeat, we performed standard checks on the residuals of the models using the DHARMa package in R (ref. ^100^), including the following tests: Kolmogorov–Smirnov for correct distribution, dispersion and outliers. Kolmogorov–Smirnov deviation was significant for six repeats in the changing-niche model (numbers 1, 4, 24, 37, 52 and 56) and five repeats for the fixed-niche model (numbers 1, 4, 24, 37 and 56). Outlier test deviation was significant for eight repeats of the changing-niche model (numbers 25, 38, 67, 69, 74, 78, 85 and 95) and 11 for the fixed-niche model (numbers 18, 24, 25, 30, 38, 67, 74, 77, 78, 85 and 95). All other tests and repeats were non-significant. Visual inspection of these deviations revealed that they were mostly capturing patterns of very small magnitude owing to the high power associated with the large size of the datasets (as a result of the large number of background points) and thus were judged not to affect an analysis. A comparison of models with outliers and models without outliers showed that the model effects were qualitatively similar, thus confirming that the outliers were not leading to artefactual conclusions. We also tested for spatial autocorrelation in the residuals calculating Moran’s *I* (ref. ^101^) with the same package. Moran’s *I* values were statistically significant (*P*-value < 0.05) but negligible (<0.01 on a scale between 0 and 1) in all cases (Supplementary Table 3).

#### Model evaluation

**Extended Data Fig. 7:**
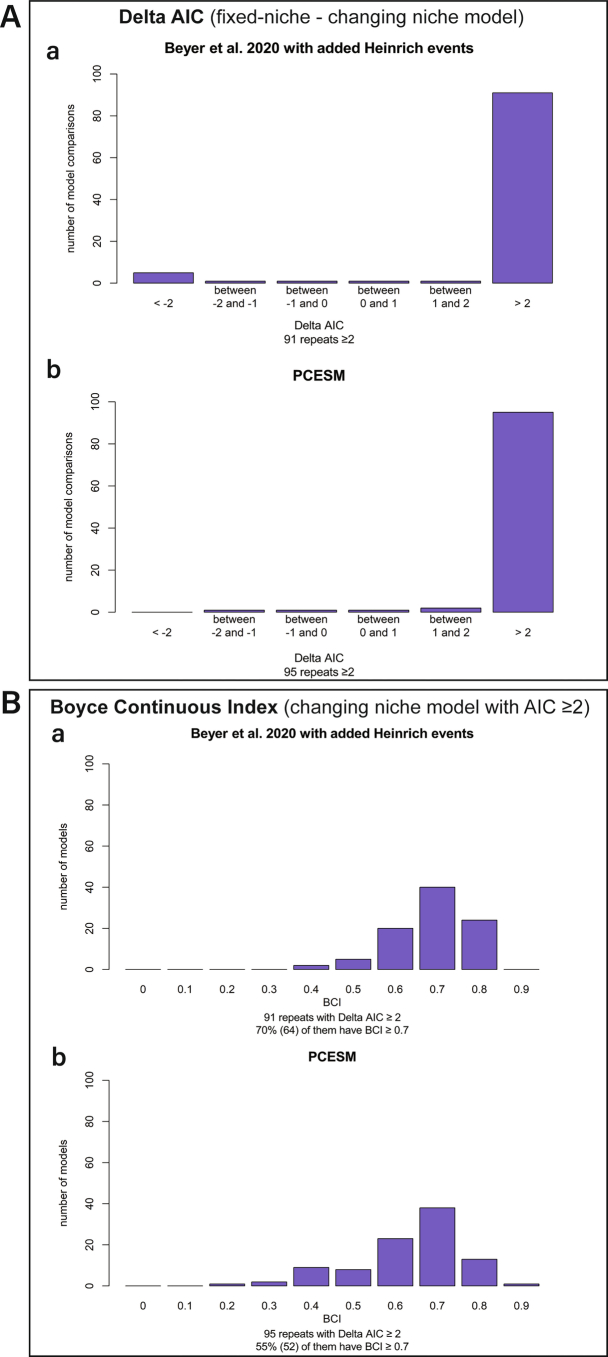
Histogram of delta AIC and BCI. **A**, Histogram of delta AIC between the fixed-niche versus changing-niche model in the 100 repeats. A score ≥ 2 supports the time-varying-niche model. **a**, Beyer et al.^57^ with added Heinrich events, 91 repeats have a delta AIC ≥ 2. **b**, PCESM^58,59^, 95 repeats have a delta AIC ≥ 2. **B**, BCI for the changing-niche model. The BCI has only been calculated on the repeats having an AIC ≥ 2, hence supporting the changing-niche model. These have been used to build the ensembles. **a**, Beyer et al.^57^ with added Heinrich events, 91 repeats with AIC ≥ 2; among them, 70% (64) have BCI ≥ 0.7. **b**, PCESM^58,59^, 95 repeats with AIC ≥ 2; among them, 55% (52) have BCI ≥ 0.7.

For each repeat, we compared the fixed-niche and changing-niche models using the AIC and found strong support for the latter in all cases (Extended Data Fig. 7a). We then evaluated the fit of the changing-niche models with the BCI^102^, which is specifically designed to be used with presence-only data, setting an acceptance threshold of Pearson’s correlation coefficient > 0.7 (ref. ^67^) (Extended Data Fig. 7b).

#### Ensemble

To achieve more robust predictions^66^, we calculated the mean and median ensembles across the 100 repeats in the following way. We selected only repeats that had an AIC > 2, hence strongly supporting the changing-niche model (91/100 for Beyer et al.^62^ with Heinrich events, 95/100 for PCESM^63,64^; Extended Data Fig. 7a). From them, we kept only repeats with a BCI > 0.7 (which accounted for 70% for Beyer et al.^62^ with Heinrich events and 55% for PCESM^63,64^; Extended Data Fig. 7b). From this selection of repeats, we created four ensembles (two for each palaeoclimatic model) by computing the mean and median of the predictions. Then, to estimate the goodness of fit of each ensemble, we computed the mean and median of the BCI estimated for each of their individual repeats (note that each repeat had a different set of occurrences and background points as a result of the random resampling).

#### Visualizing potential distribution through time (Fig. 3)

As the mean ensemble performed better than the one based on the median, it was used to visualize how the potential distribution of humans changed through time. To incorporate the uncertainty associated with the model, the predicted probabilities of occurrence were transformed into binary (presence or absence) using three different thresholds. The first one (darkest colour in Fig. 3) represents the minimum predicted area encompassing 90% of our occurrences and identifies the ‘core area’ suitable for humans (that is, the space most likely occupied). We similarly plotted a ‘peripheral area’ encompassing 95% and the ‘total area’ encompassing 99% of occurrences. This was done using a modified version of the function ecospat.mpa() from the ecospat R package^103^, in which no rounding of the digit is performed. To better visualize how the reconstructed potential distribution changed through time, we calculated for each climatic period the mean of the projections from the individual time slides. A similar procedure has been applied to the fixed-niche models, (using only the repeats with AIC < 2), for which the equivalent of Fig. 3 is available as Extended Data Fig. 2).

#### Visualizing niche changes through time (Extended Data Figs. 3 and 9)

To visualize the changing impact of different BIO variables through time in the niche-changing model, we used the function draw.gam() from the R package gratia^104^ following Leonardi et al.^65^. This function plots the interactions between time and each of the BIO variables. The result is a heat map with time represented on the *x* axis and the values of the variable of interest on the *y* axis. Within the plot, the surface colour (‘partial effect’) shows the effect on the probability of occurrence: red means an increased and blue a decreased probability compared with what would be expected on the basis of the mean value of other variables (the black lines are isoclines to aid reading the changes in the surface). We created partial effect surfaces for all repeats included in each ensemble and then plotted the mean surface, which captures consistent signals across all randomized sets of background points (Extended Data Fig. 3 for Beyer et al.^62^ with added Heinrich events and Extended Data Fig. 9 for PCESM^63,64^).

#### Analysing the SDM output (script s13)

The analyses described in this section were performed on the regions combining the core and peripheral areas (that is, dark and medium purple in Fig. 3; from now on: suitable habitat). For each time slice, we subdivided the suitable habitat into three different classes of biomes (forest, savannah and desert) based on BIOME4 (ref. ^62^) reconstructions. Ideally, we would have done it for the finer subdivisions provided by the original model, but the limited number of occurrences in the archaeological record prevents us from performing any formal analysis at this disaggregated level (for example, we only have two points for tropical rainforest). The list of biomes included in each class can be found in Supplementary Table 4. We then aggregated the results by MIS by keeping for each cell the most common biome class within the given period (Fig. 4a).

To visualize how much of the suitable area falls into each biome class (Fig. 4b), we performed the following steps. First, we summarized the five BIO climatic variables by computing the first two principal components, pooling all land cells for all of the time steps (that is, creating a principal component analysis that represents the full climatic space of Africa for the whole period considered in this research). Then, in that 2D space, for each biome class and time step, we calculated the area covered by the 2D kernel that includes 99% of the suitable cells (core and peripheral areas) within that biome class^105^. We repeated this procedure separately for the changing-niche and fixed-niche models, which are presented in Extended Data Fig. 4).

#### Testing against different palaeoenvironmental simulations (script s16)

**Extended Data Fig. 8:**
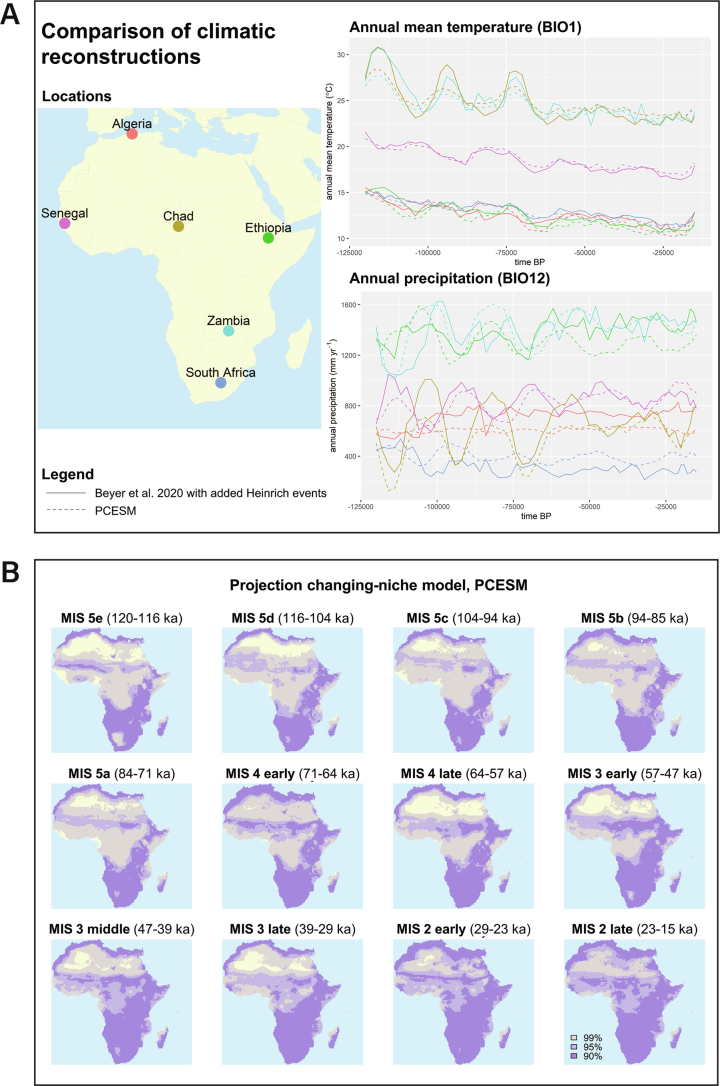
Comparison of climate simulations and projection of the changing niche through time using PCESM^58,59^ climate simulations. **a**, Comparison of the Beyer et al.^57^ with added Heinrich events (continuous lines) and PCESM^58,59^ (dashed lines) simulations. Mean annual temperature (BIO 1) and total annual precipitation (BIO 12) for six African locations are shown. **b**, Projection of the changing-niche model through time (using climate simulations from PCESM^58,59^). The core (90% of occurrences), peripheral (95% of occurrences) and total area (99% of occurrences) are shown.

**Extended Data Fig. 9:**
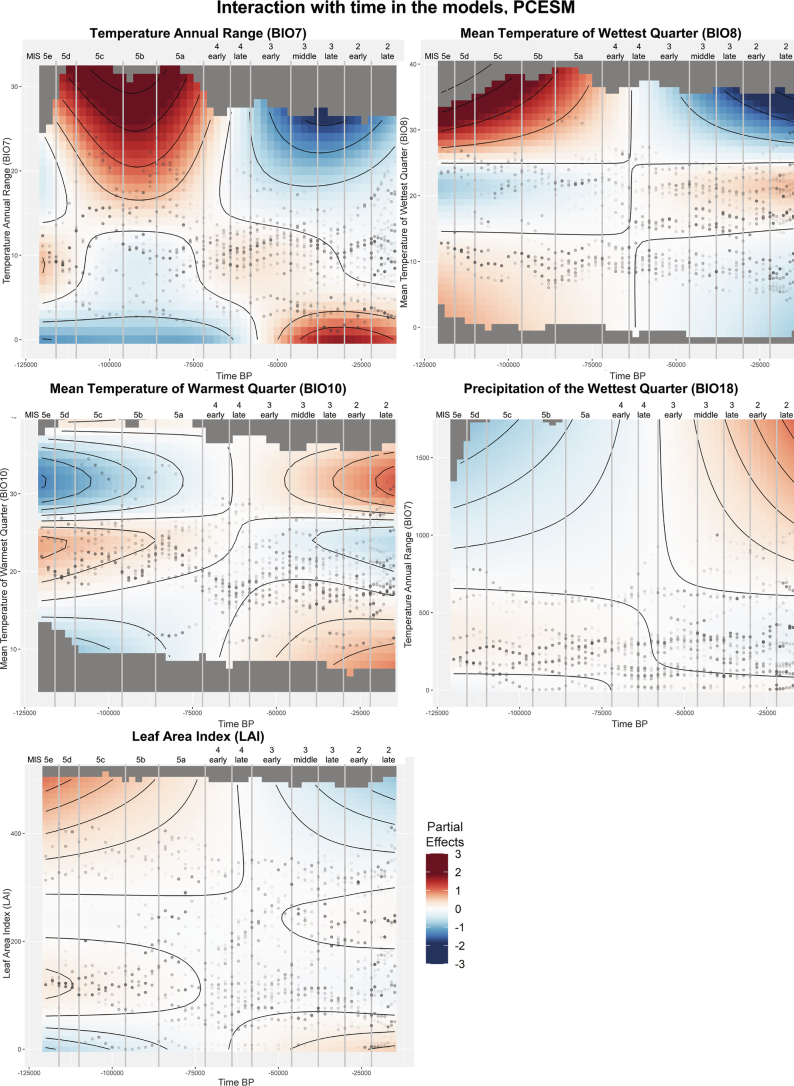
Interaction with time in the models built using the PCESM^58,59^ climatic simulations. *Homo sapiens* resampled occurrences are shown as grey dots (overlapping points are shown in darker shades based on their number). The partial effects of the GAMs (*z* axis, colour shading) show the interaction between the variable of interest (*y* axis) and time (*x* axis). Here we present the mean values of the partial effects plots over 100 repeats. The red shading represents favourable variable space (used more than expected by chance given the availability in the whole continent) and blue represents less favourable variable space. The variables used in the GAMs are: temperature annual range (BIO 7), mean temperature of the wettest quarter (BIO 8), mean temperature of the warmest quarter (BIO 10), precipitation of the wettest quarter (BIO 16) and LAI.

**Extended Data Fig. 10:**
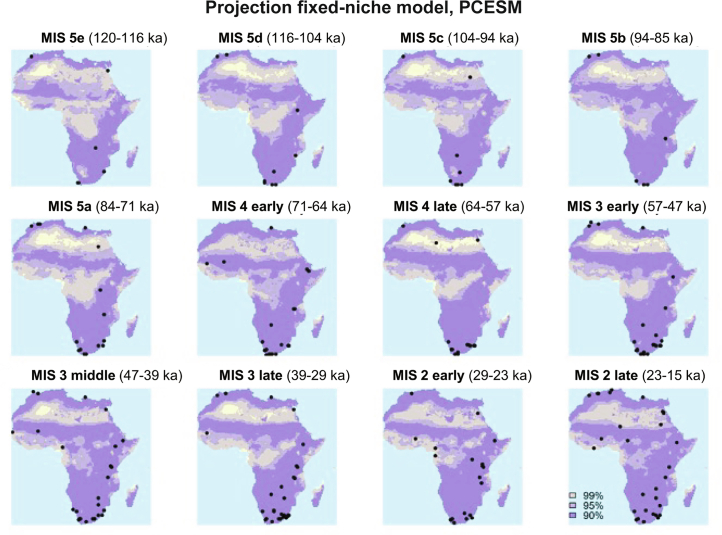
Projection of the fixed-niche model through time (using climate simulations from PCESM^58,59^. The core (90% of occurrences), peripheral (95% of occurrences) and total area (99% of occurrences).

To correct for the potential biases linked to the specific palaeoclimatic simulations used, we repeated the whole set of analyses described (excluding the standard checks on the residuals and the calculation of Moran’s *I*) with the PCESM^63,64^ climatic simulations. The AIC and BCI are comparable with the analyses presented here (Extended Data Fig. 7a,b), as are the patterns in the response surfaces of the GAMs, in which we consistently observe a change during MIS 4 (Extended Data Fig. 9). Projections of the fixed-niche and changing-niche models and results of the biome analyses are available as Extended Data Figs. 8b, 4 and 10).

#### Figure design

The cartographic representation of Africa in Fig. 1 was originally developed on QGIS 3.22 Białowieża with WGS 84 projection. Geolocated archaeological layers were superimposed and colour-coded on the basis of chronology (Fig. 1a). Overlapping sites in Fig. 1a were stacked in ascending chronological order, with older sites overlapping younger sites. A map with archaeological occurrence/layer density per square kilometre (Fig. 1b) was developed using an automated density cluster function on QGIS 3.22 Białowieża. The resulting cartographic representations were exported as .svg format and composed on Adobe Illustrator 2025. Figures 2, 3 and 4 originated as .svg files from R. Cartographic panels were processed in Adobe Photoshop 2023 and subsequently designed and composed using Adobe Illustrator 2023.

### Reporting summary

Further information on research design is available in the Nature Portfolio Reporting Summary linked to this article.

## Online content

Any methods, additional references, Nature Portfolio reporting summaries, source data, extended data, supplementary information, acknowledgements, peer review information; details of author contributions and competing interests; and statements of data and code availability are available at 10.1038/s41586-025-09154-0.

## Supplementary information

Supplementary Information Supplementary Text sections 1 and 2, full descriptions of Supplementary Tables 1–4 (tables supplied separately) and Supplementary References.

Reporting Summary

Supplementary Table 1 Database of radiometrically dated Pleistocene occupation layers from archaeological sites in Africa used in this study; see Supplementary Information document for full description.

Supplementary Table 2 Calibration of ^14^C radiocarbon ages; see Supplementary Information document for full description.

Supplementary Table 3 Tests of spatial autocorrelation in the residuals of the GAM with Moran’s *I* values; see Supplementary Information document for full description.

Supplementary Table 4 Biome classifications from Beyer et al.^62^ with added Heinrich events and from PCESM; see Supplementary Information document for full description.

## Data Availability

Climate data extracted from refs. ^62–64^. Archaeological site data from ref. ^84^. All archaeological data included in this study are cited and provided in the Supplementary Information. All palaeoclimate data included in this study are cited and provided in the Supplementary Information. Palaeoclimate data are archived and available at https://figshare.com/s/2c4253aead69d37268a8.

## Code availability

Code to perform all analyses in this study is available at the following links: script 1: https://figshare.com/s/d7de3f42aa9e2423bfb5; script 2: https://figshare.com/s/291f499a2b7907dfb62b; scripts s01 to s16: https://figshare.com/s/3570c73e7a1d6a5e783e.
