# Supplementary material for: Major expansion in the human niche preceded out of Africa dispersal
Source: Nature. 2025 Jun 18;644(8075):115–21. doi: 10.1038/s41586-025-09154-0 (PMC12328235; doi:10.1038/s41586-025-09154-0)
Supplement: Supplementary file 1 — Supplementary Text sections 1 and 2, full descriptions of Supplementary Tables 1–4 (tables supplied separately) and Supplementary References. [file 41586_2025_9154_MOESM1_ESM.pdf]

nature portfolio

https://doi.org/10.1038/s41586-025-09154-0

Supplementary information

Major expansion in the human niche preceded out of Africa dispersal

In the format provided by the authors and unedited

Major expansion in the human niche preceded out of Africa dispersal

Supplementary Information

Supplementary Information Guide

SI-1 Human Origins: Fossils and Archaeology brief overview
SI-2 Dispersals within and out of Africa
Supplementary Table 1
Supplementary Table 2
Supplementary Table 3
Supplementary Table 4
Supplementary References

SI-1 Human Origins: Fossils and Archaeology brief overview

Homo sapiens originated in Africa during the Middle Pleistocene (781-126 thousand years ago or ka). This is supported by fossil, genetic, and archaeological evidence. In Africa, the later part of the Middle Pleistocene corresponds to the archaeological period known as the Middle Stone Age (MSA). The associated fossil record is somewhat sparse. At present the first appearance date of "early or recent anatomically modern" *H. sapiens*[106] is 315 ± 34 ka[107] at Jebel Irhoud, Morocco, in association with MSA technology. The Jebel Irhoud fossils have a modern human facial and dental morphology, but a more archaic braincase morphology[106]. Hublin et al.[106] suggest that by about 300 ka, the human facial morphology had been established, while the braincase continued to evolve more recently in *H. sapiens* evolutionary history. The earliest *H. sapiens* fossil that exhibits a modern braincase morphology was recently re-dated to a minimum age of 233 ± 22 ka[108] (previously 155-187 ka[109]) and was found in Ethiopia[110-112]. The Omo Kibish 2 fossils include skull fragments and postcranial bones[113-115]. Similarly robust anatomically modern human fossils dating to ~160 ka were also found at Herto, Middle Awash, Ethiopia[116]. Anatomically modern human fossils have been directly dated at Qafzeh, Israel to 100±5 ka[117]. Additional anatomically modern human fragmented cranial and postcranial fossils of a similar age have been recovered at Klasies River in South Africa[118]. The cranial and mandibular remains from Klasies River fossils include gracile individuals[119] as well as robust individuals lacking a pronounced mental eminence[120]. The postcranial remains from Klasies River suggest affinities with both archaic and recent human populations[121]. Cranial fragments of anatomically modern human fossils have also been recovered in Ethiopia at Aduma and are dated to ~100 ka[122]. All these fossils consist of a mix of different archaic and modern features[123], and it

is not until between 100 and 40 ka that the full constellation of features that defines humans today is found in single individuals[124].

The archaeological record in Africa underwent a profound shift with the emergence of Middle Stone Age (MSA) technology from around >300 ka. The shift in focus from shaping a block of raw material to removing stone flakes from the block resulted in an explosion of new tool types, not previously seen in the African record, and long thought to be a hallmark of modern cognition[125]. The discovery of very early MSA technology with some of the earliest fossils of *H. sapiens* at Jebel Irhoud has since indicated a link between the biological and behavioral manifestations of MSA humans. Generic MSA technology is not dissimilar to Eurasian Middle Palaeolithic technology in that basic elements include forms of scrapers, retouched points, and denticulates often made using both Levallois and non-Levallois reduction methods. MSA assemblages are generally characterized by featuring a higher frequency and morphological variability of unifacial and bifacial points, as well as basally thinned pieces, which mark them out from Middle Palaeolithic assemblages[126].

The composition of the MSA flaked stone industries remained largely unchanged until about 130 ka, after which there is remarkable regional and temporal variability. The MSA is followed by Later Stone Age (LSA) technologies, with the earliest evidence of this shift in some regions from around 40 ka[127], but perhaps as early as 67 ka[128]. The transition between the MSA and LSA over Africa is spatio-temporally complex and not a uniform and parallel process (see Bader et al.[129]). However, relatively brief periods of a marked increase in the frequency of complex material culture and behavioral innovation within the MSA are evident in several regions of Africa, most notably the northern and southern ends of the continent. Many of these innovations do not appear to have had significant continuity and were used to greater and lesser degrees over time that more reflect a complex mosaic rather than a cumulative process[34]. This notwithstanding, the MSA witnessed a series of key developments, including the emergence of long-distance social networks and the transport of exotic raw material[48], bow and arrow technology[130], traps and snares[131], water storage[72], personal ornaments[26,27,30,132,133], geometric engravings[72,134], drawings[135], and stone tool technology that was regionally distinctive both in form[136,137] and methods of hafting[138-140].

In southern Africa, a proliferation of traits associated with innovative MSA human behavior appear first between ~100-70 ka. At Blombos Cave, an "ochre processing workshop" was identified from deposits dated to 100 ka[37], pierced shell beads were recovered from deposits dated to ~75 ka[27], by ~75 ka pressure flaked stone tools are present[141], and bone tool technology is present by ~100 ka at Klasies River Main site Cave 1A[142] and occurs more regularly by ~72 ka at Blombos Cave[143,144] and at Sibudu Cave[145]. At Pinnacle Point 5-6, heat treatment of silcrete to improve stone tool manufacture is present by ~72 ka[36], microlithic technology is present by ~71 ka[35], and dense shell middens[146] are present in sediments dated to 70.6 ± 2.3 ka[35]. Regionally distinct bone tools have been identified at Sibudu Cave and are from deposits dated to ~72 ka

and ~64-57 ka[145,147]. Long-term use of diverse marine resources and settlement of coastal landscapes is well-established in southern but also northern Africa between at least ~160-50 ka[148].

In northern Africa, innovative MSA technologies also appear beginning ~100 ka. The collection and use of shells as personal ornaments (eg. *Nassarius* sp. and *Tritia* sp.) appear throughout coastal and inland Morocco ~115 ka[30]. Pierced *Nassarius* sp. shell beads *in situ* have been identified at Bizmoune and may date to ≥142 ka[133], at Contrebandiers Cave dated to ~115-96 ka[149-151], at El Harhoura 2 dated to ~116-100 ka[152], at El Mnasra dated to 108-106 ka[41,152], at Ifri n'Ammar dated to ~83.3 ka[132,153], and at Taforalt from deposits dated to ~82.5 ka[26]. A "bone knife" tool from Dar es-Soltan I cave was identified in Aterian deposits dated to ~90 ka ago[154], and recently available chronologies at El Mnasra estimate the age of bone tool[155-157]-bearing Aterian layers to be ~107 ka[41]. A worked bone industry was identified at Contrebandiers Cave in deposits dated to 120-90 ka[158].

In northern Africa, the region closest to the land route into Eurasia, cultural changes peaked around the Last Interglacial, the time when early human fossils are also found in the Levant. The basic Middle Palaeolithic (MP) technology in the Levant during this time was not substantially different than the MSA in neighbouring regions of Africa[159]. Repeated humid episodes in the Saharo-Arabian arid belt from around 125 ka likely facilitated these dispersals, as well as widespread expansions within the Saharan region itself[160] and now arid areas of the Arabian Peninsula[161,162]. However, iconic innovations, such as Aterian tanged tools do not appear to cross the Nile[126]. By the beginning of Marine Isotope Stage 4, (MIS 4, 71-57 ka), widespread aridification appears to have driven the contraction or extirpation of many populations in this region[126]. For example, Aterian tanged tool assemblages that had previously dominated vast regions of northern Africa contract to the Mediterranean coast and the central Saharan mountains, where they eventually disappear during this timeframe[32]. However by the end of MIS 4 and the beginning of MIS 3, multiple, if comparable, trajectories of technological change within the MSA are observed in northeast Africa[126], perhaps indicating a re-expansion of populations shortly before dispersal out of Africa. At Haua Fteah in Libya, the MSA material culture intermittently continues until about 46-41 ka and is then replaced by a completely different and apparently unique blade-based industry, the so-called 'Dabban' around 39 ka[85]. The Dabban post-dates the appearance of Upper Palaeolithic technologies in the Levant[163], as well as elsewhere in Europe[164,165].

By MIS 3, Later Stone Age (LSA) assemblages begin to replace MSA assemblages. The oldest examples of potential LSA technology come from eastern Africa, at Panga Ya Saidi in Kenya, where relevant assemblages have been dated to MIS 4, at around ~67 ka[128]. While the profound economic shift underlying the transition from the MSA to the LSA is not well understood, the lifeways underpinning the LSA spread rapidly throughout MIS 3, until the MSA is all but replaced in most of Africa by ~20 ka[129,166-168] (but see Scerri et al. (2021)[169]). The LSA is usually

denoted by a significant reduction in the size of stone artefacts with a focus on microlithic tools, sometimes appearing in standardized, geometric form, as well as a simplification of lithic technology and an increase in non-lithic technologies[127,170] . The LSA continued to dominate the African record until the end of the Pleistocene[171,172]. While LSA populations clearly had different practices than MSA populations and continued expanding into new regions and environments of Africa, they remained hunter-gatherers without evidence for agriculture, animal husbandry or sedentarism, and therefore shared many of the same vulnerabilities with Middle and earlier Late Pleistocene humans. For these reasons, we explore the expansion of the human niche in our study until the end of the Pleistocene.

SI-2 Dispersals within and out of Africa

The oldest MSA sites are found in eastern, southern, and north-eastern Africa[42]. Despite the similarity of the technology, the few associated human fossils are diverse, as discussed above. It seems likely that there wasn't a single, geographically circumscribed 'birthplace' for *H. sapiens*[1], but that various morphs on the *H. sapiens* lineage may have been spread out in Africa. Whether this includes most, or just parts, of Africa is currently not well understood. It is therefore difficult to reconstruct within-Africa dispersals. However, the emergence of geographically discrete and highly distinctive material cultures within the MSA, such as the Aterian and Howiesons Poort technocomplexes, coupled with the diversity of the early *H. sapiens* fossil record, suggests that mixing was not constant.

It has been suggested that some of these early populations may have left Africa relatively early. Genetic studies have suggested that some very early *H. sapiens*, or populations on the lineage leading to us, left Africa and replaced the Neanderthal mitochondrial genome, possibly before 270 kya[173,174]. Modern looking fossils from Apidima, Greece may also date to >210 ka[15], while fossil evidence at Misliya in Israel is dated to 177-194 ka[14]. Later human fossils from Skhul and Qafzeh[175-177] in the Mount Carmel of present-day Israel have yielded dates that suggest a dispersal ~125-82 ka during Marine Isotope Stage 5. A human fossil from northern Arabia was dated to the end of this range, at around 85 ka[11].

It is currently very difficult to determine whether these lines of evidence indicate multiple early dispersals, or just one or two[10]. Generally, these earlier dispersals are viewed as relating to climatic windows[81] in which Saharo-Arabia greened, thus permitting animals and humans to cross the formerly impenetrable desert barrier into Eurasia. If the dates and taxonomic identifications are correct for Apidima and Misliya, it would seem that dispersal took place during interglacial MIS 7 to early glacial MIS 6. However, the age of fossil remains is not necessarily the age of dispersal, which could have happened much earlier. Whatever the case, these earlier incursions into Eurasia do not seem to have left any genetic legacy in living Eurasian populations. One of the main lines of evidence for this is the Neanderthal ancestry found in all present-day and ancient non-African modern human genomes that have been studied to date. This ancestry is mostly consistent with originating from a single episode of admixture[2,3,6,7] which had mostly ceased by 50–60 ka[4,178-180].

Supplementary Table 1. Database of radiometrically dated Pleistocene occupation layers from archaeological sites in Africa used in this study. This database includes archaeological layers that have published coordinates, radiometric dates, an error range less than or equal to 20,000 years, and a mean age ≤120,000 and ≥ 14,000 years. Bioclimatic variables for each archaeological layer were accessed from Beyer et al. (2020)[62] with added Heinrich events.

Supplementary Table 2. Calibration of 14C radiocarbon ages. This database includes the number assigned to each archaeological site name and layer for Extended Data Figure 5, as well as uncalibrated ages and errors, calibrated ages and 1σ errors that were used in the current study, and calibrated ages and 1σ errors that were not used in the current study.

Supplementary Table 3. Tests of spatial autocorrelation in the residuals of the GAM with Moran's I values. This table includes estimates for Moran's I, the expected values and their significance for both fixed-niche and changing-niche models. Each row represents a different resampling of presences and pseudo-absences.

Supplementary Table 4. Biome classifications from Beyer et al (2020)[62] with added Heinrich events and from PCESM[63,64]. Translation from the biome categories in the original publications to the threee major classes, forest, savannah and desert, as used in our analyses.

Supplementary References

106 Hublin, J.-J. *et al.* New fossils from Jebel Irhoud, Morocco and the pan-African origin of Homo sapiens. *Nature* **546**, 289-292 (2017). https://doi.org:10.1038/nature22336

107 Richter, D. *et al.* The age of the hominin fossils from Jebel Irhoud, Morocco, and the origins of the Middle Stone Age. *Nature* **546**, 293-296 (2017). https://doi.org:10.1038/nature22335

108 Vidal, C. M. *et al.* Age of the oldest known Homo sapiens from eastern Africa. *Nature* **601**, 579-583 (2022). https://doi.org:10.1038/s41586-021-04275-8

109 Aubert, M. *et al.* Confirmation of a late middle Pleistocene age for the Omo Kibish 1 cranium by direct uranium-series dating. *Journal of Human Evolution* **63**, 704-710 (2012). https://doi.org:https://doi.org/10.1016/j.jhevol.2012.07.006

110 McDougall, I., Brown, F. H. & Fleagle, J. G. Stratigraphic placement and age of modern humans from Kibish, Ethiopia. *Nature* **433**, 733-736 (2005). https://doi.org:10.1038/nature03258

111 McDougall, I., Brown, F. H. & Fleagle, J. G. Sapropels and the age of hominins Omo I and II, Kibish, Ethiopia. *Journal of Human Evolution* **55**, 409-420 (2008). https://doi.org:https://doi.org/10.1016/j.jhevol.2008.05.012

112 Brown, F. H., McDougall, I. & Fleagle, J. G. Correlation of the KHS Tuff of the Kibish Formation to volcanic ash layers at other sites, and the age of early Homo sapiens (Omo I and Omo II). *Journal of Human Evolution* **63**, 577-585 (2012). https://doi.org:https://doi.org/10.1016/j.jhevol.2012.05.014

113 Day, M. & Stringer, C. The Omo Kibish cranial remains and classification within the genus Homo. *L'Anthropologie* **95**, 573-594 (1991).

114 Pearson, O. M., Fleagle, J. G., Grine, F. E. & Royer, D. F. Further new hominin fossils from the Kibish Formation, southwestern Ethiopia. *Journal of Human Evolution* **55**, 444-447 (2008). https://doi.org:https://doi.org/10.1016/j.jhevol.2008.05.013

115 Hammond, A. S., Royer, D. F. & Fleagle, J. G. The Omo-Kibish I pelvis. *Journal of Human Evolution* **108**, 199-219 (2017). https://doi.org:https://doi.org/10.1016/j.jhevol.2017.04.004

116 White, T. D. *et al.* Pleistocene Homo sapiens from Middle Awash, Ethiopia. *Nature* **423**, 742-747 (2003). https://doi.org:10.1038/nature01669

117 McDermott, F., Hawkesworth, C. J., Grün, R. & Stringer, C. B. Dating hominid remains. *Nature* **366**, 415-415 (1993). https://doi.org:10.1038/366415b0

118 Grine, F. E., Wurz, S. & Marean, C. W. The Middle Stone Age human fossil record from Klasies River Main Site. *Journal of Human Evolution* **103**, 53-78 (2017). https://doi.org:https://doi.org/10.1016/j.jhevol.2016.12.001

119 Rightmire, G. P. & Deacon, H. J. Comparative studies of Late Pleistocene human remains from Klasies River Mouth, South Africa. *Journal of Human Evolution* **20**, 131-156 (1991). https://doi.org:https://doi.org/10.1016/0047-2484(91)90054-Y

120 Rightmire, G. P. Middle and later Pleistocene hominins in Africa and Southwest Asia. *Proceedings of the National Academy of Sciences* **106**, 16046 (2009). https://doi.org:10.1073/pnas.0903930106

121 Churchill, S. E., Pearson, O. M., Grine, F. E., Trinkaus, E. & Holliday, T. W. Morphological affinities of the proximal ulna from Klasies River main site: archaic or modern? *Journal of Human Evolution* **31**, 213-237 (1996). https://doi.org:https://doi.org/10.1006/jhev.1996.0058

122 Haile-Selassie, Y., Asfaw, B. & White, T. D. Hominid cranial remains from upper pleistocene deposits at Aduma, Middle Awash, Ethiopia. *American Journal of Physical Anthropology* **123**, 1-10 (2004). https://doi.org:https://doi.org/10.1002/ajpa.10330

123 Stringer, C. The origin and evolution of Homo sapiens. *Philosophical Transactions of the Royal Society B: Biological Sciences* **371**, 20150237 (2016). https://doi.org:10.1098/rstb.2015.0237

124 Gunz, P. *et al.* Early modern human diversity suggests subdivided population structure and a complex out-of-Africa scenario. *Proceedings of the National Academy of Sciences* **106**, 6094-6098 (2009). https://doi.org:10.1073/pnas.0808160106

125 Barham, L. in *Social Brain, Distributed Mind* (eds R. Dunbar, C. Gamble, & J. Gowlett) 367–389 (Oxford University Press, 2010).

126 Scerri, E. M. L. & Spinapolice, E. E. Lithics of the North African Middle Stone Age: assumptions, evidence and future directions. *Journal of Anthropological Sciences* **97**, 9-43 (2019). https://doi.org:doi 10.4436/jass.97002

127 Villa, P. *et al.* Border Cave and the beginning of the Later Stone Age in South Africa. *Proc Natl Acad Sci U S A* **109**, 13208-13213 (2012). https://doi.org:10.1073/pnas.1202629109

128 Shipton, C. *et al.* 78,000-year-old record of Middle and Later Stone Age innovation in an East African tropical forest. *Nature Communications* **9**, 1832 (2018). https://doi.org:10.1038/s41467-018-04057-3

129 Bader, G. D., Mabuza, A., Price Williams, D. & Will, M. Rethinking the Middle to Later Stone Age transition in southern Africa - A perspective from the highveld of Eswatini. *Quaternary Science Reviews* **286**, 107540 (2022). https://doi.org:https://doi.org/10.1016/j.quascirev.2022.107540

130 Lombard, M. & Phillipson, L. Indications of bow and stone-tipped arrow use 64 000 years ago in KwaZulu-Natal, South Africa. *Antiquity* **84**, 635-648 (2010). https://doi.org:10.1017/S0003598X00100134

131 Wadley, L. Were snares and traps used in the Middle Stone Age and does it matter? A review and a case study from Sibudu, South Africa. *Journal of Human Evolution* **58**, 179-192 (2010). https://doi.org:https://doi.org/10.1016/j.jhevol.2009.10.004

132 d'Errico, F. *et al.* Additional evidence on the use of personal ornaments in the Middle Paleolithic of North Africa. *Proceedings of the National Academy of Sciences* **106**, 16051 (2009). https://doi.org:10.1073/pnas.0903532106

133 Sehasseh, E. M. *et al.* Early Middle Stone Age personal ornaments from Bizmoune Cave, Essaouira, Morocco. *Science Advances* **7**, eabi8620 (2021). https://doi.org:10.1126/sciadv.abi8620

134 Henshilwood, C. S., d'Errico, F. & Watts, I. Engraved ochres from the Middle Stone Age levels at Blombos Cave, South Africa. *Journal of Human Evolution* **57**, 27-47 (2009). https://doi.org:10.1016/j.jhevol.2009.01.005

135 Henshilwood, C. S. *et al.* An abstract drawing from the 73,000-year-old levels at Blombos Cave, South Africa. *Nature* **562**, 115-118 (2018). https://doi.org:10.1038/s41586-018-0514-3

136 Mackay, A., Stewart, B. A. & Chase, B. M. Coalescence and fragmentation in the late Pleistocene archaeology of southernmost Africa. *Journal of Human Evolution* **72**, 26-51 (2014). https://doi.org:https://doi.org/10.1016/j.jhevol.2014.03.003

137 Will, M., Bader, G. D. & Conard, N. J. Characterizing the Late Pleistocene MSA Lithic Technology of Sibudu, KwaZulu-Natal, South Africa. *PLoS One* **9**, e98359 (2014). https://doi.org:10.1371/journal.pone.0098359

138 Lombard, M. & Pargeter, J. Hunting with Howiesons Poort segments: pilot experimental study and the functional interpretation of archaeological tools. *Journal of Archaeological Science* **35**, 2523-2531 (2008). https://doi.org:https://doi.org/10.1016/j.jas.2008.04.004

139 Scerri, E. M. L. On the spatial and technological organisation of hafting modifications in the North African Middle Stone Age. *Journal of Archaeological Science* **40**, 4234-4248 (2013). https://doi.org:https://doi.org/10.1016/j.jas.2013.06.011

140 Rots, V., Lentfer, C., Schmid, V. C., Porraz, G. & Conard, N. J. Pressure flaking to serrate bifacial points for the hunt during the MIS5 at Sibudu Cave (South Africa). *PLoS One* **12**, e0175151 (2017). https://doi.org:10.1371/journal.pone.0175151

141 Mourre, V., Villa, P. & Henshilwood, C. S. Early Use of Pressure Flaking on Lithic Artifacts at Blombos Cave, South Africa. *Science* **330**, 659-662 (2010). https://doi.org:10.1126/science.1195550

142 Bradfield, J. & Wurz, S. A functional assessment of the notched bone artefacts from Klasies River Main site. *South African Archaeological Bulletin* **75**, 128-136 (2020).

143 Henshilwood, C. S., d'Errico, F., Marean, C. W., Milo, R. G. & Yates, R. An early bone tool industry from the Middle Stone Age at Blombos Cave, South Africa: implications for the origins of modern human behaviour, symbolism and language. *Journal of Human Evolution* **41**, 631-678 (2001). https://doi.org:10.1006/jhev.2001.0515

144 d'Errico, F. & Henshilwood, C. S. Additional evidence for bone technology in the southern African Middle Stone Age. *Journal of Human Evolution* **52**, 142-163 (2007). https://doi.org:10.1016/j.jhevol.2006.08.003

145 d'Errico, F., Backwell, L. R. & Wadley, L. Identifying regional variability in Middle Stone Age bone technology: The case of Sibudu Cave. *Journal of Archaeological Science* **39**, 2479-2495 (2012). https://doi.org:10.1016/j.jas.2012.01.040

146 Marean, C. W. The origins and significance of coastal resource use in Africa and Western Eurasia. *Journal of Human Evolution* **77**, 17-40 (2014). https://doi.org:https://doi.org/10.1016/j.jhevol.2014.02.025

147 Backwell, L., d'Errico, F. & Wadley, L. Middle Stone Age bone tools from the Howiesons Poort layers, Sibudu Cave, South Africa. *Journal of Archaeological Science* **35**, 1566-1580 (2008). https://doi.org:https://doi.org/10.1016/j.jas.2007.11.006

148 Will, M., Kandel, A. W. & Conard, N. J. Midden or Molehill: The Role of Coastal Adaptations in Human Evolution and Dispersal. *Journal of World Prehistory* **32**, 33-72 (2019). https://doi.org:10.1007/s10963-018-09127-4

149 Jacobs, Z. *et al.* Single-grain OSL dating at La Grotte des Contrebandiers (‘Smugglers’ Cave’), Morocco: improved age constraints for the Middle Paleolithic levels. *Journal of Archaeological Science* **38**, 3631-3643 (2011). https://doi.org:https://doi.org/10.1016/j.jas.2011.08.033

150 Dibble, H. L. *et al.* New Excavations at the Site of Contrebandiers Cave, Morocco. *Paleoanthropology* **2012**, 145-201 (2012). https://doi.org:doi:10.4207/PA.2012.ART74

151 Aldeias, V., Goldberg, P., Dibble, H. L. & El-Hajraoui, M. Deciphering site formation processes through soil micromorphology at Contrebandiers Cave, Morocco. *Journal of Human Evolution* **69**, 8-30 (2014). https://doi.org:10.1016/j.jhevol.2013.12.016

152 Campmas, E. *et al.* Were Upper Pleistocene human/non-human predator occupations at the Témara caves (El Harhoura 2 and El Mnasra, Morocco) influenced by climate change? *Journal of Human Evolution* **78**, 122-143 (2015).

153 Richter, D., Moser, J., Nami, M., Eiwanger, J. & Mikdad, A. New chronometric data from Ifri n’Ammar (Morocco) and the chronostratigraphy of the Middle Palaeolithic in the Western Maghreb. *Journal of Human Evolution* **59**, 672-679 (2010). https://doi.org:https://doi.org/10.1016/j.jhevol.2010.07.024

154 Bouzouggar, A. *et al.* 90,000 year-old specialised bone technology in the Aterian Middle Stone Age of North Africa. *PLoS One* **13**, e0202021 (2018). https://doi.org:10.1371/journal.pone.0202021

155 El Hajraoui, M. in *Mediterrâneo* Vol. 2 105-121 (1993).

156 El Hajraoui, M. A. L'industrie osseuse atérienne de la grotte d'El Mnasra (Région de Témara, Maroc). *Préhistoire Anthropologie Méditerranéennes, LAPMO Université de Provence CNRS* **3**, 91-94 (1994).
157 El Hajraoui, M. & Debénath, A. in *Villes et sites d'archéologie Marocaine, Royaume du Maroc* Vol. 3 (eds M. A. El Hajraoui, A. Debénath, R. Nespoulet, & H. L. Dibble) Ch. El Mnasra-Chapter XXIV, 179-188 (2012).
158 Hallett, E. Y. *et al.* A worked bone assemblage from 120,000–90,000 year old deposits at Contrebandiers Cave, Atlantic Coast, Morocco. *iScience* **24**, 102988 (2021). https://doi.org:https://doi.org/10.1016/j.isci.2021.102988
159 Groucutt, H. S., Scerri, E. M. L., Stringer, C. & Petraglia, M. D. Skhul lithic technology and the dispersal of Homo sapiens into Southwest Asia. *Quaternary International* **515**, 30-52 (2019). https://doi.org:https://doi.org/10.1016/j.quaint.2017.12.027
160 Scerri, E. M. L., Drake, N. A., Jennings, R. & Groucutt, H. S. Earliest evidence for the structure of Homo sapiens populations in Africa. *Quaternary Science Reviews* **101**, 207-216 (2014). https://doi.org:https://doi.org/10.1016/j.quascirev.2014.07.019
161 Groucutt, H. S. & Petraglia, M. D. The prehistory of the Arabian peninsula: Deserts, dispersals, and demography. *Evolutionary Anthropology: Issues, News, and Reviews* **21**, 113-125 (2012). https://doi.org:https://doi.org/10.1002/evan.21308
162 Nicholson, S. L., Hosfield, R., Groucutt, H. S., Pike, A. W. G. & Fleitmann, D. Beyond arrows on a map: The dynamics of Homo sapiens dispersal and occupation of Arabia during Marine Isotope Stage 5. *Journal of Anthropological Archaeology* **62**, 101269 (2021). https://doi.org:https://doi.org/10.1016/j.jaa.2021.101269
163 Rebollo, N. R. *et al.* New radiocarbon dating of the transition from the Middle to the Upper Paleolithic in Kebara Cave, Israel. *Journal of Archaeological Science* **38**, 2424-2433 (2011). https://doi.org:https://doi.org/10.1016/j.jas.2011.05.010
164 Benazzi, S. *et al.* Early dispersal of modern humans in Europe and implications for Neanderthal behaviour. *Nature* **479**, 525-528 (2011). https://doi.org:10.1038/nature10617
165 Hublin, J.-J. *et al.* Initial Upper Palaeolithic Homo sapiens from Bacho Kiro Cave, Bulgaria. *Nature (London)* **581**, 299-302 (2020). https://doi.org:10.1038/s41586-020-2259-z
166 Marks, A. & Conard, N. in *Space and Time: Which Diachronies, Which Synchronies, Which Scales? Typology Vs. Technology* (eds T. Aubry, F. Almeida, A. C. Araújo, & M. Tiffagom) 123-131 (BAR International Series, 2008).
167 Doerschner, N. *et al.* A New Chronology for Rhafas, Northeast Morocco, Spanning the North African Middle Stone Age through to the Neolithic. *PLoS One* **11**, e0162280 (2016). https://doi.org:10.1371/journal.pone.0162280
168 Bousman, C. B. & Brink, J. S. The emergence, spread, and termination of the Early Later Stone Age event in South Africa and southern Namibia. *Quaternary International* **495**, 116-135 (2018). https://doi.org:https://doi.org/10.1016/j.quaint.2017.11.033
169 Scerri, E. M. L. *et al.* Continuity of the Middle Stone Age into the Holocene. *Scientific Reports* **11**, 70 (2021). https://doi.org:10.1038/s41598-020-79418-4
170 Scerri, E. (Oxford University Press, 2017).
171 Barham, L. & Mitchell, P. *The First Africans: African Archaeology from the Earliest Toolmakers to Most Recent Foragers*. (Cambridge University Press, 2008).
172 Mitchell, P. *The Archaeology of Southern Africa*. 2 edn, (Cambridge University Press, 2024).
173 Meyer, M. *et al.* Nuclear DNA sequences from the Middle Pleistocene Sima de los Huesos hominins. *Nature* **531**, 504-507 (2016). https://doi.org:10.1038/nature17405

174 Posth, C. *et al.* Deeply divergent archaic mitochondrial genome provides lower time boundary for African gene flow into Neanderthals. *Nature Communications* **8**, 16046 (2017). https://doi.org:10.1038/ncomms16046
175 Mercier, N. *et al.* Thermoluminescence Date for the Mousterian Burial Site of Es-Skhul, Mt. Carmel. *Journal of Archaeological Science* **20**, 169-174 (1993). https://doi.org:https://doi.org/10.1006/jasc.1993.1012
176 Stringer, C. B., Grün, R., Schwarcz, H. P. & Goldberg, P. ESR dates for the hominid burial site of Es Skhul in Israel. *Nature* **338**, 756-758 (1989). https://doi.org:10.1038/338756a0
177 Schwarcz, H. P. *et al.* ESR dates for the hominid burial site of Qafzeh in Israel. *Journal of Human Evolution* **17**, 733-737 (1988). https://doi.org:https://doi.org/10.1016/0047-2484(88)90063-2
178 Sankararaman, S., Patterson, N., Li, H., Pääbo, S. & Reich, D. The Date of Interbreeding between Neandertals and Modern Humans. *PLOS Genetics* **8**, e1002947 (2012). https://doi.org:10.1371/journal.pgen.1002947
179 Sankararaman, S., Mallick, S., Patterson, N. & Reich, D. The Combined Landscape of Denisovan and Neanderthal Ancestry in Present-Day Humans. *Current Biology* **26**, 1241-1247 (2016). https://doi.org:https://doi.org/10.1016/j.cub.2016.03.037
180 Moorjani, P. *et al.* A genetic method for dating ancient genomes provides a direct estimate of human generation interval in the last 45,000 years. *Proceedings of the National Academy of Sciences* **113**, 5652 (2016). https://doi.org:10.1073/pnas.1514696113
181 Osypińska, M. & Osypiński, P. Animal Exploitation and Behaviour of the Latest Middle Stone Age Societies in the Middle Nile Valley: Archaeozoological and Taphonomic Analysis of Late Pleistocene Fauna from the Affad Basin, Sudan. *African Archaeological Review* **33**, 107-127 (2016). https://doi.org:10.1007/s10437-016-9220-4
182 Osypinski, P., Morley, M. W., Osypinska, M. & Kotarba-Morley, A. M. Affad 23: settlement structures and palaeoenvironments in the Terminal Pleistocene of the Middle Nile Valley, Sudan. *Antiquity* **90**, 894-913 (2016).
183 Brandt, S. A. The Upper Pleistocene and early Holocene prehistory of the Horn of Africa. *African Archaeological Review* **4**, 41-82 (1986). https://doi.org:10.1007/BF01117035
184 Clark, J. D. & Williams, M. A. J. Recent Archaeological Research in Southeastern Ethiopia. 1974 - 1975. *Annales d'Éthiopie*, 19-44 (1978).
185 Chenorkian, R. Ivory Coast Prehistory: recent developments. *African Archaeological Review* **1**, 127-142 (1983). https://doi.org:10.1007/BF01116775
186 Bon, F. *et al.* Prehistory of the Central Main Ethiopian Rift (Ziway-Shala basin): Establishing the Late Stone Age sequence in Eastern Africa. *Annales d'Éthiopie* **28**, 405-407 (2013).
187 Close, A. E. Current Research and Recent Radiocarbon Dates from Northern Africa. *The Journal of African History* **21**, 145-167 (1980).
188 Sari, L. Diachronic Variation in Microlith Production Systems During the Late Pleistocene, Algeria. *African Archaeological Review*, 1-33 (2020).
189 Vermeersch, P. M. in *The Mediterranean from 50,000 to 25,000 BP: Turning Points and New Directions* (eds Marta Camps & C. Szmidt) 67–88 (Oxbow Books, 2008).
190 Wendorf, F. & Schild, R. *Prehistory of the Nile valley*. (Academic Press, 1976).
191 Chevrier, B. *et al.* Between continuity and discontinuity: An overview of the West African Paleolithic over the last 200,000 years. *Quaternary International* **466**, 3-22 (2018). https://doi.org:https://doi.org/10.1016/j.quaint.2017.11.027
192 Yeshurun, R. Taphonomy of old archaeofaunal collections: New site-formation and subsistence data for the Late Paleolithic Nile Valley. *Quaternary International* **471**, 35-54 (2018). https://doi.org:https://doi.org/10.1016/j.quaint.2017.06.027

193 Vermeersch, P. M. & Van Neer, W. Nile behaviour and Late Palaeolithic humans in Upper Egypt during the Late Pleistocene. *Quaternary Science Reviews* **130**, 155-167 (2015). https://doi.org:https://doi.org/10.1016/j.quascirev.2015.03.025

194 Pleurdeau, D. *et al.* Cultural change or continuity in the late MSA/Early LSA of southeastern Ethiopia? The site of Goda Buticha, Dire Dawa area. *Quaternary International* **343**, 117-135 (2014). https://doi.org:https://doi.org/10.1016/j.quaint.2014.02.001

195 Tribolo, C. *et al.* Across the Gap: Geochronological and Sedimentological Analyses from the Late Pleistocene-Holocene Sequence of Goda Buticha, Southeastern Ethiopia. *PLoS One* **12**, e0169418 (2017). https://doi.org:10.1371/journal.pone.0169418

196 Reade, H., O'Connell, T. C., Barker, G. & Stevens, R. E. Pleistocene and Holocene palaeoclimates in the Gebel Akhdar (Libya) estimated using herbivore tooth enamel oxygen isotope compositions. *Quaternary International* **404**, 150-162 (2016). https://doi.org:https://doi.org/10.1016/j.quaint.2015.10.009

197 Klein, R. G. & Scott, K. Re-analysis of faunal assemblages from the Haua Fteah and other late quaternary archaeological sites in Cyrenaican Libya. *Journal of Archaeological Science* **13**, 515-542 (1986). https://doi.org:https://doi.org/10.1016/0305-4403(86)90038-5

198 Barker, G. *et al.* The Cyrenaican Prehistory Project 2008: the second season of investigations of the Haua Fteah cave and its landscape, and further results from the initial (2007) fieldwork. *Libyan Studies* **39**, 175-221 (2008). https://doi.org:10.1017/S0263718900010074

199 Eiwanger, J., Mikdad, A., Moser, J. & Nami, M. Découverte de coquilles perforées de type Nassarius au site Ifri n'Ammar (Rif Oriental, Maroc). *Bulletin d'Archéologie Marocaine* **22**, 9-15 (2012).

200 Moser, J. *La Grotte d'Ifri n'Ammar, Vol. 1: L'Ibéromaurusien*. 180 (Linden Soft, 2003).

201 Nami, M. & Moser, J. *La Grotte d'Ifri n'Ammar, Tome 2: Le Paléolithique Moyen*. (Reichert Verlag, 2010).

202 Kurashina, H. *An Examination of Prehistoric Lithic Technology in East-Central Ethiopia*, University of California, Berkeley, (1978).

203 Brandt, S. A. *et al.* Early MIS 3 occupation of Mochena Borago Rockshelter, Southwest Ethiopian Highlands: Implications for Late Pleistocene archaeology, paleoenvironments and modern human dispersals. *Quaternary International* **274**, 38-54 (2012). https://doi.org:https://doi.org/10.1016/j.quaint.2012.03.047

204 Brandt, S., Hildebrand, E., Vogelsang, R., Wolfhagen, J. & Wang, H. A new MIS 3 radiocarbon chronology for Mochena Borago Rockshelter, SW Ethiopia: Implications for the interpretation of Late Pleistocene chronostratigraphy and human behavior. *Journal of Archaeological Science: Reports* **11**, 352-369 (2017). https://doi.org:https://doi.org/10.1016/j.jasrep.2016.09.013

205 Mercader, J. & Marti, R. in *Under the Canopy: The Archaeology of Tropical Rain Forests* (ed J. Mercader) 64-92 (Rutgers University Press, 2002).

206 Mercader, J., Martí, R., Martínez, J. L. & Brooks, A. The nature of 'stone-lines' in the African Quaternary record: archaeological resolution at the rainforest site of Mosumu, Equatorial Guinea. *Quaternary International* **89**, 71-96 (2002). https://doi.org:https://doi.org/10.1016/S1040-6182(01)00082-9

207 Clark, J. D., Williamson, K. D., Michels, J. W. & Marean, C. A. A Middle Stone Age occupation site at Porc Epic Cave, Dire Dawa (east-central Ethiopia). *African Archaeological Review* **2**, 37-71 (1984). https://doi.org:10.1007/BF01117225

208 Oakley, K., Campbell, B. & Molleson, T. *Catalogue of Fossil Hominids. Part I: Africa*. (British Museum of Natural History, 1967).

209 Pleurdeau, D. Human Technical Behavior in the African Middle Stone Age: The Lithic Assemblage of Porc-Epic Cave (Dire Dawa, Ethiopia). *African Archaeological Review* **22**, 177-197 (2005). https://doi.org:10.1007/s10437-006-9000-7

210 Rosso, D. E., d'Errico, F. & Zilhão, J. Stratigraphic and spatial distribution of ochre and ochre processing tools at Porc-Epic Cave, Dire Dawa, Ethiopia. *Quaternary International* **343**, 85-99 (2014). https://doi.org:https://doi.org/10.1016/j.quaint.2013.10.019

211 Cornelissen, E. On Microlithic Quartz Industries at the End of the Pleistocene in Central Africa: The Evidence from Shum Laka (NW Cameroon). *African Archaeological Review* **20**, 1-24 (2003). https://doi.org:10.1023/A:1022830321377

212 Moeyersons, J. Geomorphological processes and their palaeoenvironmental significance at the Shum Laka cave (Bamenda, western Cameroon). *Palaeogeography, Palaeoclimatology, Palaeoecology* **133**, 103-116 (1997). https://doi.org:https://doi.org/10.1016/S0031-0182(96)00148-4

213 Mercier, N. *et al.* Thermoluminescence Dating of a Middle Palaeolithic Occupation at Sodmein Cave, Red Sea Mountains (Egypt). *Journal of Archaeological Science* **26**, 1339-1345 (1999). https://doi.org:https://doi.org/10.1006/jasc.1998.0369

214 Moeyersons, J., Vermeersch, P. M. & Van Peer, P. Dry cave deposits and their palaeoenvironmental significance during the last 115ka, Sodmein Cave, Red Sea Mountains, Egypt. *Quaternary Science Reviews* **21**, 837-851 (2002). https://doi.org:https://doi.org/10.1016/S0277-3791(01)00132-9

215 Moeyersons, J. *et al.* in *Aspects of African Archaeology* (eds G. Pwiti & R. Soper) 53-62 (University of Zimbabwe Publications, 1996).

216 Van Peer, P., Vermeersch, P., Moeyersons, J. & Van Neer, W. in *Aspects of African Archaeology* (eds G. Pwiti & R. Soper) 149-156 (University of Zimbabwe Publications, 1996).

217 Hogue, J. T. & Barton, R. N. E. New radiocarbon dates for the earliest Later Stone Age microlithic technology in Northwest Africa. *Quaternary International* **413**, 62-75 (2016). https://doi.org:https://doi.org/10.1016/j.quaint.2015.11.144

218 Merzoug, S. *Comportements de subsistance des Ibéromaurusiens d'après l'analyse archéozoologique des mammifères des sites de Tamar Hat, Taza 1 et Columnata (Algérie)*, Paris, Muséum national d'histoire naturelle, (2005).

219 Merzoug, S. Essai d'interprétation du statut économique du Megaceroides algericus durant l'Ibéromaurusien dans le massif des Babors (Algérie). *Quaternaire. Revue de l'Association française pour l'étude du Quaternaire* **23**, 3 (2012).

220 Merzoug, S. & Sari, L. Re-examination of the Zone I Material from Tamar Hat (Algeria): Zooarchaeological and Technofunctional Analyses. *African Archaeological Review* **25**, 57-73 (2008). https://doi.org:10.1007/s10437-008-9028-y

221 Sari, L. & Kim, K.-J. Lithic Economy and Specialized Activities Among the Iberomaurusian Populations of Tamar Hat Rockshelter (Northeastern Algeria). *African Archaeological Review* **34**, 543-556 (2017). https://doi.org:10.1007/s10437-017-9274-y

222 Banks, K. M., Snortland, S., Cummings, L. S., Gatto, M. C. & Usai, D. The Terminal Late Palaeolithic in Wadi Kubbaniya, Egypt. *Antiquity* **89**, 346 (2015).

223 Van Peer, P., Vermeersch, P. M. & Paulissen, E. *Chert quarrying, lithic technology and a modern human burial at the palaeolithic site of Taramsa 1, Upper Egypt*. Vol. 5 (Leuven University Press, 2010).

224 Vermeersch, P. M. *et al.* A Middle Palaeolithic burial of a modern human at Taramsa Hill, Egypt. *Antiquity* **72**, 475-484 (1998).

225 Tribolo, C. *et al.* Thermoluminescence dating of a Stillbay–Howiesons Poort sequence at Diepkloof Rock Shelter (Western Cape, South Africa). *Journal of Archaeological Science* **36**, 730-739 (2009). https://doi.org:https://doi.org/10.1016/j.jas.2008.10.018

226 Vogelsang, R. *Middle-Stone-Age-Fundstellen in Südwest-Namibia*. (Heinrich-Barth-Institut, 1998).

227 Vogelsang, R. *et al.* New Excavations of Middle Stone Age Deposits at Apollo 11 Rockshelter, Namibia: Stratigraphy, Archaeology, Chronology and Past Environments. *Journal of African Archaeology* **8**, 185-218 (2010).

228 Watts, I. *The Origin of Symbolic Culture: The Middle Stone Age of Southern Africa and Khoisan Ethnography*. (University of London, 1998).

229 Wendt, W. E. 'Art Mobilier' from the Apollo 11 Cave, South West Africa: Africa's Oldest Dated Works of Art. *The South African Archaeological Bulletin* **31**, 5-11 (1976). https://doi.org:10.2307/3888265

230 Thackeray, J. F. An Analysis of Faunal Remains from Archaeological Sites in Southern South West Africa (Namibia). *The South African Archaeological Bulletin* **34**, 18-33 (1979). https://doi.org:10.2307/3888168

231 Deacon, H. J. Excavations at Boomplaas cave - a sequence through the upper Pleistocene and Holocene in South Africa. *World Archaeology* **10**, 241-257 (1979). https://doi.org:10.1080/00438243.1979.9979735

232 Klein, R. G. A Preliminary Report on the Larger Mammals from the Boomplaas Stone Age Cave Site, Cango Valley, Oudtshoorn District, South Africa. *The South African Archaeological Bulletin* **33**, 66-75 (1978). https://doi.org:10.2307/3888252

233 Pargeter, J., Loftus, E., Mackay, A., Mitchell, P. & Stewart, B. New ages from Boomplaas Cave, South Africa, provide increased resolution on late/terminal Pleistocene human behavioural variability. *Azania: Archaeological Research in Africa* **53**, 156-184 (2018). https://doi.org:10.1080/0067270X.2018.1436740

234 Watts, I. Ochre in the Middle Stone Age of Southern Africa: Ritualised Display or Hide Preservative? *The South African Archaeological Bulletin* **57**, 1-14 (2002). https://doi.org:10.2307/3889102

235 Vogel, J. C. in *Humanity from African Naissance to Coming Millennia Colloquia in Human Biology and Palaeoanthropology* (eds Phillip V Tobias, M. A. Raath, Jacopo Moggi-Cecchi, & Gerald A. Doyle) 261-268 (Firenze University Press; Witwatersrand University Press, 2001).

236 Beaumont, P. B., Miller, G.H., & Vogel, J. C. Contemplating old clues to the impact of future greenhouse climates in South Africa. *South African Journal of Science* **88**, 490-498 (1992). https://doi.org:doi:10.10520/AJA00382353_9913

237 Bird, M. I. *et al.* Radiocarbon dating from 40 to 60kaBP at Border Cave, South Africa. *Quaternary Science Reviews* **22**, 943-947 (2003). https://doi.org:https://doi.org/10.1016/S0277-3791(03)00005-2

238 Butzer, K. W., Beaumont, P. B. & Vogel, J. C. Lithostratigraphy of Border Cave, KwaZulu, South Africa: a Middle Stone Age sequence beginning c. 195,000 b.p. *Journal of Archaeological Science* **5**, 317-341 (1978). https://doi.org:https://doi.org/10.1016/0305-4403(78)90052-3

239 d'Errico, F. & Backwell, L. Earliest evidence of personal ornaments associated with burial: the Conus shells from Border Cave. *J Hum Evol* **93**, 91-108 (2016). https://doi.org:10.1016/j.jhevol.2016.01.002

240 d'Errico, F. *et al.* Early evidence of San material culture represented by organic artifacts from Border Cave, South Africa. *Proceedings of the National Academy of Sciences - PNAS* **109**, 13214-13219 (2012). https://doi.org:10.1073/pnas.1204213109

241 Grün, R. & Beaumont, P. Border Cave revisited: a revised ESR chronology. *Journal of Human Evolution* **40**, 467-482 (2001). https://doi.org:https://doi.org/10.1006/jhev.2001.0471

242 Grün, R., Beaumont, P., Tobias, P. V. & Eggins, S. On the age of Border Cave 5 human mandible. *Journal of Human Evolution* **45**, 155-167 (2003). https://doi.org:https://doi.org/10.1016/S0047-2484(03)00102-7

243 Grün, R., Beaumont, P. B. & Stringer, C. B. ESR dating evidence for early modern humans at Border Cave in South Africa. *Nature* **344**, 537-539 (1990). https://doi.org:10.1038/344537a0

244 Holloway, R. L., Broadfield, D. C. & Yuan, M. S. *The human fossil record : vol. 3, brain endocasts -- the paleoneurological evidence*. (Wiley-Liss, 2004).

245 Klein, R. G. The Mammalian Fauna from the Middle and Later Stone Age (Later Pleistocene) Levels of Border Cave, Natal Province, South Africa. *The South African Archaeological Bulletin* **32**, 14-27 (1977). https://doi.org:10.2307/3887843

246 Pfeiffer, S. & Zehr, M. K. A morphological and histological study of the human humerus from Border Cave. *Journal of Human Evolution* **31**, 49-59 (1996). https://doi.org:https://doi.org/10.1006/jhev.1996.0048

247 Wood, B. A. Wiley-Blackwell encyclopedia of human evolution. (2011).

248 Badenhorst, S. & Plug, I. N. A. The faunal remains from the middle stone age levels of Bushman Rock Shelter in South Africa. *The South African Archaeological Bulletin* **67**, 16-31 (2012).

249 Louw, A. W. Bushman Rock Shelter, Ohrigstad, Eastern Transvaal: A Preliminary Investigation, 1965. *The South African Archaeological Bulletin* **24**, 39-51 (1969). https://doi.org:10.2307/3887660

250 Plug, I. Some Research Results on the Late Pleistocene and Early Holocene Deposits of Bushman Rock Shelter, Eastern Transvaal. *The South African Archaeological Bulletin* **36**, 14-21 (1981). https://doi.org:10.2307/3888014

251 Plug, I. Bone Tools and Shell, Bone and Ostrich Eggshell Beads from Bushman Rock Shelter (BRS), Eastern Transvaal. *The South African Archaeological Bulletin* **37**, 57-62 (1982). https://doi.org:10.2307/3888676

252 Porraz, G., E *et al.* Bushman Rock Shelter (Limpopo, South Africa): A perspective from the edge of the Highveld. *South African Archaeological Bulletin* **70**, 166-179 (2015).

253 Protsch, R. & de Villiers, H. Bushman rock shelter, Origstad, Eastern Transvaal, South Africa. *Journal of Human Evolution* **3**, 387-396 (1974). https://doi.org:https://doi.org/10.1016/0047-2484(74)90201-2

254 Avery, G. in *Papers in the Prehistory of the Western Cape* (eds J. Parkington & M. Hall) 164-191 (British Archaeological Reports International, 1987).

255 Cooke, H. B. S. Notes on the faunal material from the Cave of Hearths and Kalkbank. *Prehistory of the Transvaal*, 447–453 (1962).

256 Dusseldorp, G., Lombard, M. & Wurz, S. Pleistocene Homo and the updated Stone Age sequence of South Africa. *South African Journal of Science* **109** (2013). https://doi.org:10.1590/sajs.2013/20120042

257 Klein, R. G. & Cruz-Uribe, K. in *Papers in the Prehistory of the Western Cape, South Africa* (eds R. G. Klein, K. Cruz-Uribe, J. Parkington, & M. Hall) 132-164 (BAR International Series, 1987).

258 Poggenpoel, C. in *Papers in the prehistory of the western Cape, South Africa* Vol. 332 (eds J.E. Parkington & M. Hall) 212-236 (British Archaeological Reports Oxford, 1987).

259 Volman, T. P. *The Middle Stone Age in the Southern Cape*, University of Chicago, Department of Anthropology, (1981).

260 Ames, C. J. H. *et al.* Chronostratigraphy, Site Formation, and Palaeoenvironmental Context of Late Pleistocene and Holocene Occupations at Grassridge Rock Shelter (Eastern Cape, South Africa). *Open Quaternary* **6** (2020). https://doi.org:http://doi.org/10.5334/oq.77

261 Deacon, H. J. *Where hunters gathered : a study of Holocene Stone Age people in the Eastern Cape*. (South African Archaeological Society, 1976).

262 Brooks, A. S. *et al.* Dating and Context of Three Middle Stone Age Sites with Bone Points in the Upper Semliki Valley, Zaire. *Science* **268**, 548-553 (1995). https://doi.org:10.1126/science.7725099

263 Beaumont, P. in *Guide to Archaeological Sites in the Northern Cape, McGregor Museum, Kimberley, chap Kathu* (eds P. Beaumont & D. Morris) 75-100 (1990).

264 Tryon, C. A. *et al.* Middle and Later Stone Age chronology of Kisese II rockshelter (UNESCO World Heritage Kondoa Rock-Art Sites), Tanzania. *PLoS One* **13**, e0192029 (2018). https://doi.org:10.1371/journal.pone.0192029

265 Jacobs, Z. *et al.* Ages for the Middle Stone Age of Southern Africa: Implications for Human Behavior and Dispersal. *Science* **322**, 733-735 (2008). https://doi.org:10.1126/science.1162219

266 Mackay, A. A Characterization of the MSA Stone Artefact Assemblage from the 1984 Excavations at Klein Kliphuis, Western Cape. *The South African Archaeological Bulletin* **61**, 181-188 (2006). https://doi.org:10.2307/20474926

267 Mackay, A. The Late Pleistocene archaeology of Klein Kliphuis rock shelter, Western Cape, South Africa: 2006 excavation. *The South African Archaeological Bulletin* **65**, 132-147 (2010). https://doi.org:10.2307/41408506

268 Mackay, A. Nature and significance of the Howiesons Poort to post-Howiesons Poort transition at Klein Kliphuis rockshelter, South Africa. *Journal of Archaeological Science* **38**, 1430-1440 (2011). https://doi.org:https://doi.org/10.1016/j.jas.2011.02.006

269 Mackay, A. & Welz, A. Engraved ochre from a Middle Stone Age context at Klein Kliphuis in the Western Cape of South Africa. *Journal of Archaeological Science* **35**, 1521-1532 (2008). https://doi.org:https://doi.org/10.1016/j.jas.2007.10.015

270 van Rijssen, W. J. & Avery, G. The Late Holocene Deposits at Klein Kliphuis Shelter, Cedarberg, Western Cape Province. *The South African Archaeological Bulletin* **47**, 34-43 (1992). https://doi.org:10.2307/3888990

271 Klein, R. G. Later Stone Age Faunal Samples from Heuningneskrans Shelter (Transvaal) and Leopard's Hill Cave (Zambia). *The South African Archaeological Bulletin* **39**, 109-116 (1984). https://doi.org:10.2307/3888376

272 Miller, S. F. The Age of Nachikufan Industries in Zambia. *The South African Archaeological Bulletin* **26**, 143-146 (1971). https://doi.org:10.2307/3887806

273 Goodwin, A. J. H. The Montagu Cave: A full Report of the Investigation of the Montagu Rock-shelter. *Annals of the South African Museum* **24**, 1-16 (1929).

274 Keller, C. M. *Montagu Cave in Prehistory: A Descriptive Analysis*. (University of California Press, 1973).

275 Mehlman, M. J. Mumba-Höhle revisited: The relevance of a forgotten excavation to some current issues in east African prehistory. *World Archaeology* **11**, 80-94 (1979). https://doi.org:10.1080/00438243.1979.9979751

276 Mehlman, M. J. Provenience, age and associations of archaic Homo sapiens crania from Lake Eyasi, Tanzania. *Journal of Archaeological Science* **14**, 133-162 (1987). https://doi.org:https://doi.org/10.1016/0305-4403(87)90003-3

277 Prendergast, M. E. *et al.* New Excavations at Mumba Rockshelter, Tanzania. *Journal of African Archaeology* **5**, 217 (2007). https://doi.org:https://doi.org/10.3213/1612-1651-10093

278 Mehlman, M. J. Excavations at Nasera Rock, Tanzania. *Azania: Archaeological Research in Africa* **12**, 111-118 (1977). https://doi.org:10.1080/00672707709511250

279 Mehlman, M. J. *Later Quaternary archaeological sequences in northern Tanzania* Ph.D. thesis, University of Illinois at Urbana-Champaign, (1989).

280 Cooke, C. K. Report on Excavations at Pomongwe and Tshangula Caves, Matopo Hills, Southern Rhodesia. *The South African Archaeological Bulletin* **18**, 73-151 (1963). https://doi.org:10.2307/3886481

281 Walker, N. J. Later Stone Age Research in the Matopos. *The South African Archaeological Bulletin* **35**, 19-24 (1980). https://doi.org:10.2307/3888719

282 Mackay, A., Jacobs, Z. & Steele, T. E. Pleistocene Archaeology and Chronology of Putslaagte 8 (PL8) Rockshelter, Western Cape, South Africa. *Journal of African Archaeology* **13**, 71-98 (2015). https://doi.org:https://doi.org/10.3213/2191-5784-10267

283 Clark, A. M. B. The MSA/LSA Transition in Southern Africa: New Technological Evidence from Rose Cottage Cave. *The South African Archaeological Bulletin* **52**, 113-121 (1997). https://doi.org:10.2307/3889076

284 Clark, A. M. B. Late Pleistocene Technology at Rose Cottage Cave: A Search for Modern Behavior in an MSA Context. *African Archaeological Review* **16**, 93-119 (1999). https://doi.org:10.1023/A:1021955013009

285 Harper, P. T. N. The Middle Stone Age sequences at Rose Cottage Cave: a search for continuity and discontinuity. *South African Journal of Science* **93**, 470-475 (1997). https://doi.org:10.10520/AJA00382353_89

286 Hodgskiss, T. & Wadley, L. How people used ochre at Rose Cottage Cave, South Africa: Sixty thousand years of evidence from the Middle Stone Age. *PLoS One* **12**, e0176317 (2017). https://doi.org:10.1371/journal.pone.0176317

287 Pienaar, M., Woodborne, S. & Wadley, L. Optically stimulated luminescence dating at Rose Cottage Cave. *South African Journal of Science* **104**, 65-70 (2008).

288 Plug, I. & Ronette, E. The Macrofaunal Remains from Recent Excavations at Rose Cottage Cave, Orange Free State. *The South African Archaeological Bulletin* **47**, 16-25 (1992). https://doi.org:10.2307/3888988

289 Soriano, S., Villa, P. & Wadley, L. Blade technology and tool forms in the Middle Stone Age of South Africa: the Howiesons Poort and post-Howiesons Poort at Rose Cottage Cave. *Journal of Archaeological Science* **34**, 681-703 (2007). https://doi.org:https://doi.org/10.1016/j.jas.2006.06.017

290 Valladas, H. *et al.* Thermoluminescence dating on burnt lithics from Middle Stone Age layers at Rose Cottage Cave. *South African Journal of Science* **101**, 169-174 (2005). https://doi.org:10.10520/EJC96375

291 Wadley, L. Rose Cottage Cave: Background and a Preliminary Report on the Recent Excavations. *The South African Archaeological Bulletin* **46**, 125-130 (1991). https://doi.org:10.2307/3889091

292 Wadley, L. Rose Cottage Cave: The Later Stone Age Levels with European and Iron Age Artefacts. *The South African Archaeological Bulletin* **47**, 8-12 (1992). https://doi.org:10.2307/3888986

293 Wadley, L. The Robberg Industry of Rose Cottage Cave, Eastern Free State: The Technology, Spatial Patterns and Environment. *The South African Archaeological Bulletin* **51**, 64-74 (1996). https://doi.org:10.2307/3888841

294 Wadley, L. in *Settlement Dynamics of the Middle Palaeolithic and Middle Stone Age* Vol. II (ed N. J. Conard) 23-36 (Kerns Verlag, 2004).

295 Wadley, L. E. A. & Jeannerat, C. Vegetation change in the eastern Orange Free State: the Holocene and later Pleistocene evidence from charcoal studies at Rose Cottage Cave. *South African Journal of Science* **88**, 558-563 (1992). https://doi.org:10.10520/AJA00382353_9906

296 Wadley, L. & Harper, P. Rose Cottage Cave Revisited: Malan's Middle Stone Age Collection. *The South African Archaeological Bulletin* **44**, 23-32 (1989). https://doi.org:10.2307/3888316

297 Carter, P. L., Mitchell, P. J. & Vinnicombe, P. *Sehonghong : the middle and later stone age industrial sequence at a Lesotho rock-shelter*. (British Archaeological Reports, 1988).

298 Mitchell, P. J. Understanding the MSA/LSA transition: the pre-20 000 BP assemblages from new excavations at Sehonghong:rock shelter, Lesotho. ". *Southern African Field Archaeology* **3**, 15-25 (1994). https://doi.org:10.10520/AJA10195785_49

299 Mitchell, P. J. Revisiting the Robberg: New Results and a Revision of Old Ideas at Sehonghong Rock Shelter, Lesotho. *The South African Archaeological Bulletin* **50**, 28-38 (1995). https://doi.org:10.2307/3889272

300 Mitchell, P. J. The late Quaternary of the Lesotho highlands, southern Africa: Preliminary results and future potential of ongoing research at Sehonghong shelter. *Quaternary International* **33**, 35-43 (1996). https://doi.org:https://doi.org/10.1016/1040-6182(95)00097-6

301 Opperman, H. Strathalan Cave B, north-eastern Cape Province, south Africa: Evidence for human behaviour 29,000-26,000 years ago. *Quaternary International* **33**, 45-53 (1996). https://doi.org:https://doi.org/10.1016/1040-6182(95)00096-8

302 Opperman, H. & Heydenrych, B. A 22 000 Year-Old Middle Stone Age Camp Site with Plant Food Remains from the North-Eastern Cape. *The South African Archaeological Bulletin* **45**, 93-99 (1990). https://doi.org:10.2307/3887967

303 Kaplan, J. The Umhlatuzana Rock Shelter sequence: 100,000 years of Stone Age history. *Southern African Humanities* **2**, 1-94 (1990). https://doi.org:10.10520/AJA16815564_282

304 Kaplan, J. M. 45,000 Years of Hunter-Gatherer History in Natal as Seen from Umhlatuzana Rock Shelter. *Goodwin Series* **6**, 7-16 (1989). https://doi.org:10.2307/3858128

305 Lombard, M., Wadley, L., Jacobs, Z., Mohapi, M. & Roberts, R. G. Still Bay and serrated points from Umhlatuzana Rock Shelter, Kwazulu-Natal, South Africa. *Journal of Archaeological Science* **37**, 1773-1784 (2010). https://doi.org:https://doi.org/10.1016/j.jas.2010.02.015

306 Backwell, L. R. *et al.* Multiproxy record of late Quaternary climate change and Middle Stone Age human occupation at Wonderkrater, South Africa. *Quaternary Science Reviews* **99**, 42-59 (2014). https://doi.org:https://doi.org/10.1016/j.quascirev.2014.06.017

307 Donahue, R. E., Murphy, M. L. & Robbins, L. H. Lithic Microwear Analysis of Middle Stone Age Artifacts from White Paintings Rock Shelter, Botswana. *Journal of Field Archaeology* **29**, 155-163 (2002). https://doi.org:10.2307/3181490

308 Feathers, J. K. Luminescence dating of sediment samples from White paintings Rockshelter, Botswana. *Quaternary Science Reviews* **16**, 321-331 (1997). https://doi.org:https://doi.org/10.1016/S0277-3791(96)00083-2

309 Robbins, L. H. *et al.* Archaeology, Palaeoenvironment, and Chronology of the Tsodilo Hills White Paintings Rock Shelter, Northwest Kalahari Desert, Botswana. *Journal of Archaeological Science* **27**, 1085-1113 (2000). https://doi.org:https://doi.org/10.1006/jasc.2000.0597

310 Occhietti, S., Raynal, J.-P., Pichet, P. & Lefèvre, D. Aminostratigraphie des formations littorales pléistocènes et holocènes de la région de Casablanca, Maroc. *Quaternaire* **13**, 55-64 (2002). https://doi.org:https://doi.org/10.3406/quate.2002.1703

311 Raynal, J.-P. *et al.* La Grotte des Félins, site paléolithique du Pléistocène supérieur à Dar Bouazza (Maroc). *L'Anthropologie* **112**, 182-200 (2008). https://doi.org:https://doi.org/10.1016/j.anthro.2008.02.006

312 Raynal, J.-P., Alaoui, F.-Z., Mohib, A. & Geraads, D. Préhistoire ancienne au Maroc atlantique : bilan et perspectives régionales. *Bulletin d'archéologie marocaine* **XXI**, 9-54 (2009).

313 Rhodes, E. J., Singarayer, J. S., Raynal, J. P., Westaway, K. E. & Sbihi-Alaoui, F. Z. New age estimates for the Palaeolithic assemblages and Pleistocene succession of Casablanca, Morocco. *Quaternary Science Reviews* **25**, 2569-2585 (2006). https://doi.org:https://doi.org/10.1016/j.quascirev.2005.09.010

314 Texier, J.-P., Lefèvre, D., Raynal, J.-P. & El Graoui, M. Lithostratigraphy of the littoral deposits of the last one million years in the Casablanca region (Morocco) / Lithostratigraphie des dépôts littoraux contemporains du dernier million d'années dam la région de Casablanca (Maroc). *Quaternaire* **13**, 23-41 (2002).

315 Mercader, J., Gosse, J. C., Bennett, T., Hidy, A. J. & Rood, D. H. Cosmogenic nuclide age constraints on Middle Stone Age lithics from Niassa, Mozambique. *Quaternary Science Reviews* **47**, 116-130 (2012). https://doi.org:https://doi.org/10.1016/j.quascirev.2012.05.018

316 Shea, J. J. The Middle Stone Age archaeology of the Lower Omo Valley Kibish Formation: Excavations, lithic assemblages, and inferred patterns of early Homo sapiens behavior. *Journal of Human Evolution* **55**, 448-485 (2008). https://doi.org:https://doi.org/10.1016/j.jhevol.2008.05.014

317 Barker, G. *et al.* The Cyrenaican Prehistory Project 2009: the third season of investigations of the Haua Fteah cave and its landscape, and further results from the 2007–2008 fieldwork. *Libyan Studies* **40**, 55-94 (2009). https://doi.org:10.1017/S0263718900004519

318 Chazan, M. Variability in the Middle Paleolithic of Haua Fteah, Libya. *Paléorient* **21**, 65-73 (1995).

319 Hunt, C. *et al.* Site formation processes in caves: The Holocene sediments of the Haua Fteah, Cyrenaica, Libya. *Journal of Archaeological Science* **37**, 1600-1611 (2010). https://doi.org:https://doi.org/10.1016/j.jas.2010.01.021

320 McBurney, C. B. M. *The Haua Fteah (Cyrenaica) and the Stone Age of the South-East Mediterranean*. 402 (Cambridge University Press, 1967).

321 Harvati, K. *et al.* The Later Stone Age Calvaria from Iwo Eleru, Nigeria: Morphology and Chronology. *PLoS One* **6**, e24024 (2011). https://doi.org:10.1371/journal.pone.0024024

322 Cerasoni, J. N. *et al.* Human interactions with tropical environments over the last 14,000 years at Iho Eleru, Nigeria. *iScience* **26** (2023). https://doi.org:10.1016/j.isci.2023.106153

323 Bouzouggar, A., Kozłowski, J. & Otte, M. Étude des ensembles lithiques atériens de la grotte d'El Aliya à Tanger (Maroc). *L'Anthropologie* **106**, 207-248 (2002). https://doi.org:10.1016/S0003-5521(02)01090-7

324 Howe, B. *The palaeolithic of Tangier, Morocco : excavations at Cape Ashakar, 1939-1947*. (Peabody Museum, 1967).

325 Wrinn, P. J. & Rink, W. J. ESR Dating of Tooth Enamel From Aterian Levels at Mugharet el 'Aliya (Tangier, Morocco). *Journal of Archaeological Science* **30**, 123-133 (2003). https://doi.org:https://doi.org/10.1006/jasc.2002.0813

326 Avery, D. M. Micromammals as palaeoenvironmental indicators and an interpretation of the Late Quaternary in the southern Cape Province, South Africa. *Annals of the South African Museum* **85**, 183-374 (1982).

327 Avery, G. *et al.* The 1992-1993 Excavations at the Die Kelders Middle and Later Stone Age Cave Site, South Africa. *Journal of Field Archaeology* **24**, 263-291 (1997). https://doi.org:10.2307/530685

328 Feathers, J. K. & Bush, D. A. Luminescence dating of Middle Stone Age Deposits at Die Kelders. *Journal of Human Evolution* **38**, 91-119 (2000). https://doi.org:https://doi.org/10.1006/jhev.1999.0351

329 Grine, F. E. Middle Stone Age human fossils from Die Kelders Cave 1, Western Cape Province, South Africa. *Journal of Human Evolution* **38**, 129-145 (2000). https://doi.org:https://doi.org/10.1006/jhev.1999.0353

330 Grine, F. E., Klein, R. G. & Volman, T. P. Dating, archaeology and human fossils from the Middle Stone Age levels of Die Kelders, South Africa. *Journal of Human Evolution* **21**, 363-395 (1991). https://doi.org:https://doi.org/10.1016/0047-2484(91)90113-A

331 Klein, R. G. & Cruz-Uribe, K. Middle and Later Stone Age large mammal and tortoise remains from Die Kelders Cave 1, Western Cape Province, South Africa. *Journal of Human Evolution* **38**, 169-195 (2000). https://doi.org:https://doi.org/10.1006/jhev.1999.0355

332 Marean, C. W., Goldberg, P., Avery, G., Grine, F. E. & Klein, R. G. Middle Stone Age Stratigraphy and Excavations at Die Kelders Cave 1 (Western Cape Province, South Africa): the 1992, 1993, and 1995 Field Seasons. *Journal of Human Evolution* **38**, 7-42 (2000). https://doi.org:https://doi.org/10.1006/jhev.1999.0349

333 Schwarcz, H. P. & Rink, W. J. ESR dating of the Die Kelders Cave 1 Site, South Africa. *Journal of Human Evolution* **38**, 121-128 (2000). https://doi.org:https://doi.org/10.1006/jhev.1999.0352

334 Thackeray, A. I. Middle Stone Age artefacts from the 1993 and 1995 excavations of Die Kelders Cave 1, South Africa. *Journal of Human Evolution* **38**, 147-168 (2000). https://doi.org:https://doi.org/10.1006/jhev.1999.0354

335 Klein, R. G. The Mammalian Fauna of the Klasies River Mouth Sites, Southern Cape Province, South Africa. *The South African Archaeological Bulletin* **31**, 75-98 (1976). https://doi.org:10.2307/3887730

336 Singer, R. & Wymer, J. *The Middle Stone Age at Klasies River Mouth in South Africa*. (University of Chicago Press, 1982).

337 Wurz, S. Variability in the Middle Stone Age Lithic Sequence, 115,000–60,000 Years Ago at Klasies River, South Africa. *Journal of Archaeological Science* **29**, 1001-1015 (2002). https://doi.org:https://doi.org/10.1006/jasc.2001.0799

338 Bräuer, G., Deacon, H. J. & Zipfel, F. Comment on the new maxillary finds from Klasies River, South Africa. *Journal of Human Evolution* **23**, 419-422 (1992). https://doi.org:https://doi.org/10.1016/0047-2484(92)90089-R

339 Rightmire, G. P. & Deacon, H. J. New human teeth from Middle Stone Age deposits at Klasies River, South Africa. *Journal of Human Evolution* **41**, 535-544 (2001). https://doi.org:https://doi.org/10.1006/jhev.2001.0500

340 Rightmire, G. P., Deacon, H. J., Schwartz, J. H. & Tattersall, I. Human foot bones from Klasies River main site, South Africa. *Journal of Human Evolution* **50**, 96-103 (2006). https://doi.org:https://doi.org/10.1016/j.jhevol.2005.08.010

341 Grine, F. E. & Henshilwood, C. S. Additional human remains from Blombos Cave, South Africa: (1999–2000 excavations). *Journal of Human Evolution* **42**, 293-302 (2002). https://doi.org:https://doi.org/10.1006/jhev.2001.0525

342 Grine, F. E., Henshilwood, C. S. & Sealy, J. C. Human remains from Blombos Cave, South Africa: (1997–1998 excavations). *Journal of Human Evolution* **38**, 755-765 (2000). https://doi.org:https://doi.org/10.1006/jhev.1999.0379

343 Henshilwood, C. S. *et al.* Blombos Cave, Southern Cape, South Africa: Preliminary Report on the 1992–1999 Excavations of the Middle Stone Age Levels. *Journal of Archaeological Science* **28**, 421-448 (2001). https://doi.org:https://doi.org/10.1006/jasc.2000.0638

344 Jacobs, Z., Duller, G. A., Wintle, A. G. & Henshilwood, C. S. Extending the chronology of deposits at Blombos Cave, South Africa, back to 140 ka using optical dating of single and multiple grains of quartz. *J Hum Evol* **51**, 255-273 (2006). https://doi.org:10.1016/j.jhevol.2006.03.007

345 Jacobs, Z., Jones, B. G., Cawthra, H. C., Henshilwood, C. S. & Roberts, R. G. The chronological, sedimentary and environmental context for the archaeological deposits at Blombos Cave, South Africa. *Quaternary Science Reviews* **235**, 105850 (2020). https://doi.org:https://doi.org/10.1016/j.quascirev.2019.07.032
346 Tribolo, C., Mercier, N., Selo, M., Valladas, H., Joron, J-L, Reyss, J-L, Henshilwood, C., Sealy, J., Yates, R. TL dating of burnt lithics from Blombos cave (South Africa): further evidence for the antiquity of modern human behaviour. *Archaeometry* **48**, 341-357 (2006). https://doi.org:https://doi.org/10.1111/j.1475-4754.2006.00260.x
347 Vanhaeren, M., d'Errico, F., van Niekerk, K. L., Henshilwood, C. S. & Erasmus, R. M. Thinking strings: additional evidence for personal ornament use in the Middle Stone Age at Blombos Cave, South Africa. *Journal of human evolution* **64**, 500-517 (2013). https://doi.org:10.1016/j.jhevol.2013.02.001
348 Barham, L. S. The Mumbwa Caves Project, Zambia, 1993-94. *Nyame Akuma* **43**, 66 - 72 (1995).
349 Barham, L. & Debenham, N. in *The Middle Stone Age of Zambia, South Central Africa* (ed L. Barham) 43-50 (Western Academic & Specialist Press, 2000).
350 Barham, L., Pinto, A. C. & Andrews, P. in *The Middle Stone Age of Zambia, South Central Africa* 81-148 (Western Academic & Specialist Press, 2000).
351 Clark, J. D. Further excavations (1939) at the Mumbwa Caves, Northern Rhodesia. *Transactions of the Royal Society of South Africa* **29**, 133-201 (1942). https://doi.org:10.1080/00359194209519812
352 Dart, R. A. & del Grande, N. The ancient iron-smelting cavern at Mumbwa. *Transactions of the Royal Society of South Africa* **19**, 379-427 (1930).
353 Bouzouggar, A. *Matières premières, processus de fabrication et de gestion des supports d'outils dans la séquence atérienne de la grotte d'El Mnasra I (ancienne grotte des Contrebandiers) à Témara (Maroc)*, (1997).
354 Linstädter, J., Eiwanger, J., Mikdad, A. & Weniger, G.-C. Human occupation of Northwest Africa: A review of Middle Palaeolithic to Epipalaeolithic sites in Morocco. *Quaternary International* **274**, 158-174 (2012). https://doi.org:https://doi.org/10.1016/j.quaint.2012.02.017
355 Nespoulet, R. *et al.* Le contexte archéologique des restes humains atériens de la région de Rabat-Témara (Maroc): apport des fouilles des grottes d'El Mnasra et d'El Harhoura 2. *Actes des quatrièmes rencontres des Quaternaristes Marocains (RQM4), Le Quaternaire marocain dans son contexte méditerranéen*, 356-375 (2008).
356 Roche, J. Chronostratigraphie des restes atériens de la Grotte des Contrebandiers à Témara (Province de Rabat). *Bulletins et Mémoires de la Société d'Anthropologie de Paris*, 165-173 (1976).
357 Schwenninger, J.-L. *et al.* in *South-Eastern Mediterranean Peoples between 130,000 and 10,000 Years Ago* (ed Elena A.A. Garcea) 18-36 (Oxbow Books, 2010).
358 Berger, L. R. & Parkington, J. E. A new pleistocene hominid-bearing locality at Hoedjiespunt, South Africa. *American Journal of Physical Anthropology* **98**, 601-609 (1995). https://doi.org:https://doi.org/10.1002/ajpa.1330980415
359 Churchill, S. E., Berger, L. & Parkington, J. A Middle Pleistocene human tibia from Hoedjiespunt, Western Cape, South Africa. *South African Journal of Science* **96**, 367-368 (2000).
360 Stynder, D. D., Moggi-Cecchi, J., Berger, L. R. & Parkington, J. E. Human mandibular incisors from the late Middle Pleistocene locality of Hoedjiespunt 1, South Africa. *Journal of Human Evolution* **41**, 369-383 (2001). https://doi.org:https://doi.org/10.1006/jhev.2001.0488
361 Will, M., Parkington, J. E., Kandel, A. W. & Conard, N. J. Coastal adaptations and the Middle Stone Age lithic assemblages from Hoedjiespunt 1 in the Western Cape, South

Africa. *Journal of Human Evolution* **64**, 518-537 (2013). https://doi.org:https://doi.org/10.1016/j.jhevol.2013.02.012

362 Bluszcz, A. in *Egypt During the Last Interglacial: The Middle Paleolithic of Bir Tarfawi and Bir Sahara East* 224-226 (Springer US, 1993).

363 Campbell, A. L. in *Egypt During the Last Interglacial: The Middle Paleolithic of Bir Tarfawi and Bir Sahara East* 519-527 (Springer US, 1993).

364 Close, A. & Wendorf, F. in *Egypt During the Last Interglacial* (eds F. Wendorf, R. Schild, & A. Close) 473-487 (Plenum, 1993).

365 Królik, H. in *Egypt During the Last Interglacial: The Middle Paleolithic of Bir Tarfawi and Bir Sahara East* 512-518 (Springer US, 1993).

366 Królik, H. in *Egypt During the Last Interglacial: The Middle Paleolithic of Bir Tarfawi and Bir Sahara East* 528-538 (Springer US, 1993).

367 Nicoll, K. A revised chronology for Pleistocene paleolakes and Middle Stone Age@ Middle Paleolithic cultural activity at Bîr Tirfawi@ Bîr Sahara in the Egyptian Sahara. *Quaternary International* **463**, 18-28 (2018).

368 Schild, R. & Wendorf, F. in *Egypt During the Last Interglacial: The Middle Paleolithic of Bir Tarfawi and Bir Sahara East* 15-65 (Springer US, 1993).

369 Schwarcz, H. P. & Morawska, L. in *Egypt During the Last Interglacial: The Middle Paleolithic of Bir Tarfawi and Bir Sahara East* 205-217 (Springer US, 1993).

370 Wendorf, F. & Close, A. E. in *Egypt During the Last Interglacial: The Middle Paleolithic of Bir Tarfawi and Bir Sahara East* 488-511 (Springer US, 1993).

371 Wendorf, F., Schild, R. & Close, A. E. in *Egypt During the Last Interglacial: The Middle Paleolithic of Bir Tarfawi and Bir Sahara East* 552-573 (Springer US, 1993).

372 Wendorf, F. *et al.* The Prehistory of the Egyptian Sahara. *Science* **193**, 103-114 (1976). https://doi.org:10.1126/science.193.4248.103

373 Close, A. E. in *Egypt During the Last Interglacial: The Middle Paleolithic of Bir Tarfawi and Bir Sahara East* 288-344 (Springer US, 1993).

374 Close, A. E. & Wendorf, F. in *Egypt During the Last Interglacial: The Middle Paleolithic of Bir Tarfawi and Bir Sahara East* 261-264 (Springer US, 1993).

375 Hill, C. L. in *Egypt During the Last Interglacial: The Middle Paleolithic of Bir Tarfawi and Bir Sahara East* 443-458 (Springer US, 1993).

376 Hill, C. L. in *Egypt During the Last Interglacial: The Middle Paleolithic of Bir Tarfawi and Bir Sahara East* 412-423 (Springer US, 1993).

377 Hill, C. L. in *Egypt During the Last Interglacial: The Middle Paleolithic of Bir Tarfawi and Bir Sahara East* 424-442 (Springer US, 1993).

378 Hill, C. L. in *Egypt During the Last Interglacial: The Middle Paleolithic of Bir Tarfawi and Bir Sahara East* 459-470 (Springer US, 1993).

379 Huxtable, J. in *Egypt During the Last Interglacial: The Middle Paleolithic of Bir Tarfawi and Bir Sahara East* 227-228 (Springer US, 1993).

380 Królik, H. in *Egypt During the Last Interglacial: The Middle Paleolithic of Bir Tarfawi and Bir Sahara East* 399-411 (Springer US, 1993).

381 Królik, H. in *Egypt During the Last Interglacial: The Middle Paleolithic of Bir Tarfawi and Bir Sahara East* 373-379 (Springer US, 1993).

382 McKinney, C. R. in *Egypt During the Last Interglacial: The Middle Paleolithic of Bir Tarfawi and Bir Sahara East* 218-223 (Springer US, 1993).

383 Saunders, J. in *Egypt During the Last Interglacial: The Middle Paleolithic of Bir Tarfawi and Bir Sahara East* 359-372 (Springer US, 1993).

384 Schwarcz, H. P. & Grün, R. in *Egypt During the Last Interglacial: The Middle Paleolithic of Bir Tarfawi and Bir Sahara East* 234-237 (Springer US, 1993).

385 Wendorf, F. in *Egypt During the Last Interglacial: The Middle Paleolithic of Bir Tarfawi and Bir Sahara East* 345-355 (Springer US, 1993).

386 Wendorf, F. & Schild, R. in *Egypt During the Last Interglacial: The Middle Paleolithic of Bir Tarfawi and Bir Sahara East* 380-398 (Springer US, 1993).

387 Steele, T. E. & Klein, R. G. The Middle and Later Stone Age faunal remains from Diepkloof Rock Shelter, Western Cape, South Africa. *Journal of Archaeological Science* **40**, 3453-3462 (2013). https://doi.org:https://doi.org/10.1016/j.jas.2013.01.001

388 Verna, C., Texier, P.-J., Rigaud, J.-P., Poggenpoel, C. & Parkington, J. The Middle Stone Age human remains from Diepkloof Rock Shelter (Western Cape, South Africa). *Journal of Archaeological Science* **40**, 3532-3541 (2013). https://doi.org:https://doi.org/10.1016/j.jas.2013.04.011

389 Tribolo, C. *et al.* OSL and TL dating of the Middle Stone Age sequence at Diepkloof Rock Shelter (South Africa): a clarification. *Journal of Archaeological Science* **40**, 3401-3411 (2013). https://doi.org:https://doi.org/10.1016/j.jas.2012.12.001

390 Barham, L. *et al.* The dating and interpretation of a Mode 1 site in the Luangwa Valley, Zambia. *Journal of Human Evolution* **60**, 549-570 (2011). https://doi.org:10.1016/j.jhevol.2010.12.003

391 Casey, J. *et al.* Report of investigations at the Birimi site in Northern Ghana. *Nyame Akuma* **48**, 32-38 (1997).

392 Quickert, N., Godfrey-Smith, D. & Casey, J. Optical and thermoluminescence dating of Middle Stone Age and Kintampo bearing sediments at Birimi, a multi-component archaeological site in Ghana. *Quaternary Science Reviews* **22**, 1291-1297 (2003). https://doi.org:10.1016/S0277-3791(03)00050-7

393 Lebrun, B. *et al.* Establishing a West African chrono-cultural framework: First luminescence dating of sedimentary formations from the Falémé Valley, Eastern Senegal. *Journal of Archaeological Science: Reports* **7**, 379-388 (2016). https://doi.org:https://doi.org/10.1016/j.jasrep.2016.05.001

394 Wilkins, J. *et al.* Fabric Analysis and Chronology at Ga-Mohana Hill North Rockshelter, Southern Kalahari Basin: Evidence for In Situ, Stratified Middle and Later Stone Age Deposits. *Journal of Paleolithic Archaeology* **3**, 336-361 (2020). https://doi.org:10.1007/s41982-020-00050-9

395 Sereno, P. C. *et al.* Lakeside Cemeteries in the Sahara: 5000 Years of Holocene Population and Environmental Change. *PLoS One* **3**, e2995 (2008). https://doi.org:10.1371/journal.pone.0002995

396 Lukich, V., Porat, N., Faershtein, G., Cowling, S. & Chazan, M. New Chronology and Stratigraphy for Kathu Pan 6, South Africa. *Journal of Paleolithic Archaeology* **2**, 235-257 (2019). https://doi.org:10.1007/s41982-019-00031-7

397 Robert, A., Soriano, S., Rasse, M., Stokes, S. & Huysecom, E. First chrono-cultural reference framework for the West African Paleolithic: New data from Ounjougou, Dogon Country, Mali. *Journal of African Archaeology* **1**, 151-169 (2003).

398 Tribolo, C., Rasse, M., Soriano, S. & Huysecom, E. Defining a chronological framework for the Middle Stone Age in West Africa: Comparison of methods and models for OSL ages at Ounjougou (Mali). *Quaternary Geochronology* **29**, 80-96 (2015). https://doi.org:https://doi.org/10.1016/j.quageo.2015.05.013

399 Mackay, A. *et al.* Putslaagte 1 (PL1), the Doring River, and the later Middle Stone Age in southern Africa's Winter Rainfall Zone. *Quaternary International* **350**, 43-58 (2014). https://doi.org:https://doi.org/10.1016/j.quaint.2014.05.007

400 Mercier, N. *et al.* The Rhafas Cave (Morocco): Chronology of the mousterian and aterian archaeological occupations and their implications for Quaternary geochronology based on luminescence (TL/OSL) age determinations. *Quaternary Geochronology* **2**, 309-313 (2007). https://doi.org:https://doi.org/10.1016/j.quageo.2006.03.010

401 Wengler, L. La transition du Moustérien à l'Atérien. *L'Anthropologie* **101**, 448-481 (1997).

402 Wengler, L. in *Settlement Dynamics of the Middle Palaeolithic and Middle Stone Age* Vol. I (ed N. J. Conard) 65-90 (Kerns Verlag, 2001).

403 Barton, R. N. E. *et al.* Reconsidering the MSA to LSA transition at Taforalt Cave (Morocco) in the light of new multi-proxy dating evidence. *Quaternary International* **413**, 36-49 (2016). https://doi.org:https://doi.org/10.1016/j.quaint.2015.11.085

404 Barton, R. N. E. *et al.* Origins of the Iberomaurusian in NW Africa: New AMS radiocarbon dating of the Middle and Later Stone Age deposits at Taforalt Cave, Morocco. *Journal of Human Evolution* **65**, 266-281 (2013). https://doi.org:10.1016/j.jhevol.2013.06.003

405 Bouzouggar, A. *et al.* Reevaluating the Age of the Iberomaurusian in Morocco. *African Archaeological Review* **25**, 3-19 (2008). https://doi.org:10.1007/s10437-008-9023-3

406 Roche, J. La grotte de Taforalt. *L'Anthropologie* **57**, 375-380 (1953).

407 Staff, R. A. *et al.* in *Cemeteries and Sedentism in the Later Stone Age of NW Africa: Excavations at Grotte des Pigeons, Taforalt, Morocco Momographien des Römisch-Germanischen Zentralmuseums* (eds R. N. E. Barton, A. Bouzouggar, S. N. Collcutt, & L. T. Humphrey) 143-154 (Verlag des Römisch-Germanischen Zentralmuseums, 2019).

408 Taylor, V. K. *et al.* The Epipalaeolithic (Iberomaurusian) at Grotte des Pigeons (Taforalt), Morocco: A preliminary study of the land Mollusca. *Quaternary International* **244**, 5-14 (2011). https://doi.org:https://doi.org/10.1016/j.quaint.2011.04.041

409 Niang, K. *et al.* The Middle Stone Age occupations of Tiémassas, coastal West Africa, between 62 and 25 thousand years ago. *Journal of Archaeological Science: Reports* **34**, 102658 (2020). https://doi.org:https://doi.org/10.1016/j.jasrep.2020.102658

410 Barton, R. N. E., Bouzouggar, A., Collcutt, S. N., Schwenninger, J. L. & Clark-Balzan, L. OSL dating of the Aterian levels at Dar es-Soltan I (Rabat, Morocco) and implications for the dispersal of modern Homo sapiens. *Quaternary Science Reviews* **28**, 1914-1931 (2009). https://doi.org:https://doi.org/10.1016/j.quascirev.2009.03.010

411 Ruhlmann, A. La grotte préhistorique de Dar Es-Soltan, Institut des Hautes Etudes Marocaines 11. *Collections Hespéris, Paris* (1951).

412 Nespoulet, R. *et al.* Palaeolithic and Neolithic Occupations in the Témara Region (Rabat, Morocco): Recent Data on Hominin Contexts and Behavior. *African Archaeological Review* **25**, 21-39 (2008). https://doi.org:10.1007/s10437-008-9025-1

413 Stoetzel, E., Denys, C., Bailon, S., El Hajraoui, M. A. & Nespoulet, R. Taphonomic Analysis of Amphibian and Squamate Remains from El Harhoura 2 (Rabat-Témara, Morocco): Contributions to Palaeoecological and Archaeological Interpretations. *International Journal of Osteoarchaeology* **22**, 616-635 (2012). https://doi.org:https://doi.org/10.1002/oa.1275

414 Ben Arous, E., Falguères, C., Tombret, O., El Hajraoui, M. A. & Nespoulet, R. Combined US-ESR dating of fossil teeth from El Harhoura 2 cave (Morocco): New data about the end of the MSA in Temara region. *Quaternary International* (2019).

415 Campmas, E. *Caractérisation de l'occupation des sites de la région de Témara (Maroc) au Pléistocène supérieur et nouvelles données sur la subsistance des hommes du Paléolithique moyen d'Afrique du Nord: Exemples des approches taphonomiques et archéozoologiques menées sur les faunes d'El Harhoura 2 et d'El Mnasra* PhD thesis, Université Bordeaux 1, (2012).

416 El Hajraoui, M. *Le Paléolithique du domaine mésetien septentrional. Données récentes sur le littoral: Rabat, Témara et la Mamora*, Thèse de doctorat d'Etat, Université Mohamed V, Rabat, (2004).

417 Campmas, E. *et al.* Initial insights into Aterian hunter–gatherer settlements on coastal landscapes: The example of Unit 8 of El Mnasra Cave (Témara, Morocco). *Quaternary International* **413**, 5-20 (2016).

418 Churchill, S. E. *et al.* Erfkroon: A new Florisian fossil locality from fluvial contexts in the western Free State, South Africa. *South African Journal of Science* **96**, 161-163 (2000).

419 Henshilwood, C. S. *et al.* Klipdrift Shelter, southern Cape, South Africa: preliminary report on the Howiesons Poort layers. *Journal of Archaeological Science* **45**, 284-303 (2014). https://doi.org:https://doi.org/10.1016/j.jas.2014.01.033

420 Klein, R. G. The archaeological significance of animal bones from Acheulean sites in southern Africa. *African Archaeological Review* **6**, 3-25 (1988). https://doi.org:10.1007/BF01117110

421 Stewart, B. A. *et al.* Afromontane foragers of the Late Pleistocene: Site formation, chronology and occupational pulsing at Melikane Rockshelter, Lesotho. *Quaternary International* **270**, 40-60 (2012). https://doi.org:https://doi.org/10.1016/j.quaint.2011.11.028

422 Stokes, S. & Bailey, R. in *Palaeolithic Quarrying Sites in Upper and Middle Egypt* (ed Pierre M. Vermeersch) 349-350 (Leuven University Press, 2002).

423 Van Peer, P., Vermeersch, P. M., Paulissen, E. & Huyge, D. in *Palaeolithic Quarrying Sites in Upper and Middle Egypt* (ed Pierre M. Vermeersch) 139-210 (Leuven University Press, 2002).

424 Mitchell, P. J. & Steinberg, J. M. Ntloana Tsoana: A Middle Stone Age Sequence from Western Lesotho. *The South African Archaeological Bulletin* **47**, 26-33 (1992). https://doi.org:10.2307/3888989

425 Marean, C. W., Nilssen, P., Brown, K., Jerardino, A. & Stynder, D. Paleoanthropological investigations of Middle Stone Age sites at Pinnacle Point, Mossel Bay (South Africa): archaeology and hominid remains from the 2000 field season. *Paleoanthropology* **2**, 14-83 (2004).

426 Jacobs, Z. An OSL chronology for the sedimentary deposits from Pinnacle Point Cave 13B—A punctuated presence. *Journal of Human Evolution* **59**, 289-305 (2010). https://doi.org:https://doi.org/10.1016/j.jhevol.2010.07.010

427 Karkanas, P. & Goldberg, P. Site formation processes at Pinnacle Point Cave 13B (Mossel Bay, Western Cape Province, South Africa): resolving stratigraphic and depositional complexities with micromorphology. *Journal of Human Evolution* **59**, 256-273 (2010). https://doi.org:https://doi.org/10.1016/j.jhevol.2010.07.001

428 Marean, C. W. *et al.* Early human use of marine resources and pigment in South Africa during the Middle Pleistocene. *Nature* **449**, 905-908 (2007). https://doi.org:10.1038/nature06204

429 Marean, C. W. *et al.* The stratigraphy of the Middle Stone Age sediments at Pinnacle Point Cave 13B (Mossel Bay, Western Cape Province, South Africa). *Journal of Human Evolution* **59**, 234-255 (2010). https://doi.org:https://doi.org/10.1016/j.jhevol.2010.07.007

430 Thompson, E., Williams, H. M. & Minichillo, T. Middle and late Pleistocene Middle Stone Age lithic technology from Pinnacle Point 13B (Mossel Bay, Western Cape Province, South Africa). *Journal of Human Evolution* **59**, 358-377 (2010). https://doi.org:https://doi.org/10.1016/j.jhevol.2010.07.009

431 Watts, I. The pigments from Pinnacle Point Cave 13B, Western Cape, South Africa. *Journal of Human Evolution* **59**, 392-411 (2010). https://doi.org:https://doi.org/10.1016/j.jhevol.2010.07.006

432 Marean, C. W. Pinnacle Point Cave 13B (Western Cape Province, South Africa) in context: The Cape Floral kingdom, shellfish, and modern human origins. *Journal of Human Evolution* **59**, 425-443 (2010). https://doi.org:https://doi.org/10.1016/j.jhevol.2010.07.011

433 Bar-Matthews, M. *et al.* A high resolution and continuous isotopic speleothem record of paleoclimate and paleoenvironment from 90 to 53 ka from Pinnacle Point on the south

coast of South Africa. *Quaternary Science Reviews* **29**, 2131-2145 (2010). https://doi.org:https://doi.org/10.1016/j.quascirev.2010.05.009

434 Brown, K. S. *et al.* Fire As an Engineering Tool of Early Modern Humans. *Science* **325**, 859 (2009). https://doi.org:10.1126/science.1175028

435 Wilkins, J. *et al.* Lithic technological responses to Late Pleistocene glacial cycling at Pinnacle Point Site 5-6, South Africa. *PLoS One* **12**, e0174051 (2017). https://doi.org:10.1371/journal.pone.0174051

436 Allott, L. F. Archaeological charcoal as a window on palaeovegetation and wood-use during the Middle Stone Age at Sibudu Cave. *Southern African Humanities* **18**, 173-201 (2006).

437 Cain, C. R. Human activity suggested by the taphonomy of 60 ka and 50 ka faunal remains from Sibudu Cave. *Southern African Humanities* **18**, 241-260 (2006). https://doi.org:10.10520/EJC84761

438 Cochrane, G. W. G. An analysis of lithic artefacts from the ~60 ka layers of Sibudu Cave. *Southern African Humanities* **18**, 69-88 (2006). https://doi.org:10.10520/EJC84770

439 d'Errico, F., Vanhaeren, M. & Wadley, L. Possible shell beads from the Middle Stone Age layers of Sibudu Cave, South Africa. *Journal of Archaeological Science* **35**, 2675-2685 (2008). https://doi.org:https://doi.org/10.1016/j.jas.2008.04.023

440 Delagnes, A., Wadley, L., Villa, P. & Lombard, M. Crystal quartz backed tools from the Howiesons Poort at Sibudu Cave. *Southern African Humanities* **18**, 43-56 (2006). https://doi.org:10.10520/EJC84772

441 Hodgskiss, T. An investigation into the properties of the ochre from Sibudu, KwaZulu-Natal, South Africa. *Southern African Humanities* **24**, 99-120 (2012). https://doi.org:10.10520/EJC127432

442 Lombard, M. First impressions of the functions and hafting technology of Still Bay pointed artefacts from Sibudu Cave. *Southern African Humanities* **18**, 27-41 (2006).

443 Plug, I. Resource exploitation : animal use during the Middle Stone Age at Sibudu Cave, KwaZulu-Natal : Sibudu Cave. *South African Journal of Science* **100**, 151-158 (2004). https://doi.org:10.10520/EJC96239

444 Renaut, R. & Bamford, M. K. Results of a preliminary palynological analysis at Sibudu Cave. *Southern African Humanities* **18**, 235-240 (2006). https://doi.org:10.10520/EJC84762

445 Riga, A. *et al.* Human deciduous teeth from the Middle Stone Age layers of Sibudu Cave (South Africa). *Journal of Anthropological Sciences* **96**, 75-87 (2018). https://doi.org:10.4436/jass.96005

446 Sievers, C. Seeds from the Middle Stone Age layers at Sibudu Cave. *Southern African Humanities* **18**, 203-222 (2006). https://doi.org:10.10520/EJC84764

447 Villa, P. & Michel, L. Hunting weapons of the Middle Stone Age and the Middle Palaeolithic: spear points from Sibudu, Rose Cottage and Bouheben. *Southern African Humanities* **18**, 89-122 (2006).

448 Wadley, L. A Typological Study of the Final Middle Stone Age Stone Tools from Sibudu Cave, Kwazulu-Natal. *The South African Archaeological Bulletin* **60**, 51-63 (2005).

449 Wadley, L. & Jacobs, Z. Sibudu Cave: background to the excavations, stratigraphy and dating. *Southern African Humanities* **18**, 1-26 (2006).

450 Zwane, B. & Bamford, M. A reconstruction of woody vegetation, environment and wood use at Sibudu Cave, South Africa, based on charcoal that is dated between 73 and 72 ka. *Quaternary International* **593-594**, 95-103 (2021). https://doi.org:https://doi.org/10.1016/j.quaint.2020.10.026

451 Cremaschi, M., Di Lernia, S. & Garcea, E. A. A. Some Insights on the Aterian in the Libyan Sahara: Chronology, Environment, and Archaeology. *African Archaeological Review* **15**, 261-286 (1998). https://doi.org:10.1023/A:1021620531489

452 Garcea, E. A. A. *Uan Tabu in the Settlement History of the Libyan Sahara*. (All'insegna del giglio, 2001).

453 Bader, G. D., Cable, C., Lentfer, C. & Conard, N. J. Umbeli Belli Rock Shelter, a forgotten piece from the puzzle of the Middle Stone Age in KwaZulu-Natal, South Africa. *Journal of Archaeological Science: Reports* **9**, 608-622 (2016). https://doi.org:https://doi.org/10.1016/j.jasrep.2016.08.038

454 Bader, G. D., Tribolo, C. & Conard, N. J. A return to Umbeli Belli: New insights of recent excavations and implications for the final MSA of eastern South Africa. *Journal of Archaeological Science: Reports* **21**, 733-757 (2018). https://doi.org:https://doi.org/10.1016/j.jasrep.2018.08.043

455 Steele, T. E. *et al.* Varsche Rivier 003: A Middle and Later Stone Age Site with Still Bay and Howiesons Poort Assemblages in Southern Namaqualand, South Africa. *PaleoAnthropology* **2016**, 100-163 (2016).

456 Steele, T. E., Mackay, A., Orton, J. & Schwortz, S. Varsche Rivier 003, a new Middle Stone Age site in southern Namaqualand, South Africa. *The South African Archaeological Bulletin* **67**, 108–119 (2012).

457 Fisher, E. C. *et al.* Coastal occupation and foraging during the last glacial maximum and early Holocene at Waterfall Bluff, eastern Pondoland, South Africa. *Quaternary Research* **97**, 1-41 (2020). https://doi.org:10.1017/qua.2020.26

458 Thompson, J. C. *et al.* Early human impacts and ecosystem reorganization in southern-central Africa. *Science Advances* **7**, eabf9776 (2021). https://doi.org:10.1126/sciadv.abf9776

459 Debénath, A. Le peuplement préhistorique du Maroc : données récentes et problèmes. *L'Anthropologie* **104**, 131-145 (2000). https://doi.org:https://doi.org/10.1016/S0003-5521(00)90006-2

460 Debénath, A., Raynal, J.-P., Roche, J., Texier, J. P. & Ferembach, D. Stratigraphie, habitat, typologie et devenir de l'Atérien marocain : données récentes. *L'Anthropologie* **90**, 233-246 (1986).

461 Hublin, J.-J. Recent Human Evolution in Northwestern Africa. *Philosophical Transactions: Biological Sciences* **337**, 185-191 (1992).

462 Monchot, H. & Aouraghe, H. Deciphering the taphonomic history of an Upper Paleolithic faunal assemblage from Zouhrah Cave/El Harhoura 1, Morocco. *Quaternaire* **20**, 239-253 (2009).

463 Yellen, J. E., Brooks, A. S., Cornelissen, E., Mehlman, M. J. & Stewart, K. A middle stone age worked bone industry from Katanda, Upper Semliki Valley, Zaire. *Science* **268**, 553 (1995). https://doi.org:10.1126/science.7725100

464 Mercader, J. & Sillén, P. Middle and Later Stone Age sites from Sofala, Gorongosa (Central Mozambique). *Nyame Akuma* **80**, 3-13 (2013).

465 Niespolo, E. M., Sharp, W. D., Avery, G. & Dawson, T. E. Early, intensive marine resource exploitation by Middle Stone Age humans at Ysterfontein 1 rockshelter, South Africa. *Proceedings of the National Academy of Sciences* **118**, e2020042118 (2021). https://doi.org:10.1073/pnas.2020042118

466 Avery, G. *et al.* The Ysterfontein 1 Middle Stone Age Rock Shelter and the Evolution of Coastal Foraging. *Goodwin Series* **10**, 66-89 (2008).

467 Halkett, D. *et al.* First excavation of intact Middle Stone Age layers at Ysterfontein, Western Cape Province, South Africa: implications for Middle Stone Age ecology. *Journal of Archaeological Science* **30**, 955-971 (2003). https://doi.org:https://doi.org/10.1016/S0305-4403(02)00273-X

468 Klein, R. G. *et al.* The Ysterfontein 1 Middle Stone Age site, South Africa, and early human exploitation of coastal resources. *Proc Natl Acad Sci U S A* **101**, 5708 (2004). https://doi.org:10.1073/pnas.0400528101

469 Wurz, S. The significance of MIS 5 shell middens on the Cape coast: A lithic perspective from Klasies River and Ysterfontein 1. *Quaternary International* **270**, 61-69 (2012). https://doi.org:https://doi.org/10.1016/j.quaint.2011.06.032
