# Supplementary material for: Major expansion in the human niche preceded out of Africa dispersal
Source: Nature. 2025 Jun 18;644(8075):115–21. doi: 10.1038/s41586-025-09154-0 (PMC12328235; doi:10.1038/s41586-025-09154-0)
Supplement: Supplementary file 2 — Reporting Summary [file 41586_2025_9154_MOESM2_ESM.pdf]

# nature portfolio

Corresponding author(s): Emily Y. Hallett
Last updated by author(s): 4/6/2025

# Reporting Summary

Nature Portfolio wishes to improve the reproducibility of the work that we publish. This form provides structure for consistency and transparency in reporting. For further information on Nature Portfolio policies, see our Editorial Policies and the Editorial Policy Checklist.

## Statistics

For all statistical analyses, confirm that the following items are present in the figure legend, table legend, main text, or Methods section.

| n/a | Confirmed | |
|---|---|---|
| ☐ | ☒ | The exact sample size (*n*) for each experimental group/condition, given as a discrete number and unit of measurement |
| ☐ | ☒ | A statement on whether measurements were taken from distinct samples or whether the same sample was measured repeatedly |
| ☐ | ☒ | The statistical test(s) used AND whether they are one- or two-sided<br>*Only common tests should be described solely by name; describe more complex techniques in the Methods section.* |
| ☐ | ☒ | A description of all covariates tested |
| ☐ | ☒ | A description of any assumptions or corrections, such as tests of normality and adjustment for multiple comparisons |
| ☐ | ☒ | A full description of the statistical parameters including central tendency (e.g. means) or other basic estimates (e.g. regression coefficient) AND variation (e.g. standard deviation) or associated estimates of uncertainty (e.g. confidence intervals) |
| ☐ | ☒ | For null hypothesis testing, the test statistic (e.g. $F$, $t$, $r$) with confidence intervals, effect sizes, degrees of freedom and $P$ value noted<br>*Give P values as exact values whenever suitable.* |
| ☒ | ☐ | For Bayesian analysis, information on the choice of priors and Markov chain Monte Carlo settings |
| ☐ | ☒ | For hierarchical and complex designs, identification of the appropriate level for tests and full reporting of outcomes |
| ☐ | ☒ | Estimates of effect sizes (e.g. Cohen's *d*, Pearson's *r*), indicating how they were calculated |

*Our web collection on statistics for biologists contains articles on many of the points above.*

## Software and code

Policy information about availability of computer code

**Data collection**

No software was used for data collection.

**Data analysis**

Custom code to perform all analyses in this study was written in R software and is archived and available at the following links:
script 1: https://figshare.com/s/d7de3f42aa9e2423bfb5
script 2: https://figshare.com/s/291f499a2b7907dfb62b
scripts s01 to s16: https://figshare.com/s/3570c73e7a1d6a5e783e

Data analysis was performed using the following software version numbers:
QGIS: 3.22 Białowieża
Adobe Illustrator: 2025
Adobe Photoshop: 2023
R software: 4.2
rcarbon: 1.4.3
tidysdm: 0.9
pastclim: 1.2
mgcv: 1.9.0
DHARMa: 0.4.6
ecospat: 4.0
gratia: 0.7.3

For manuscripts utilizing custom algorithms or software that are central to the research but not yet described in published literature, software must be made available to editors and reviewers. We strongly encourage code deposition in a community repository (e.g. GitHub). See the Nature Portfolio guidelines for submitting code & software for further information.

## Data

Policy information about availability of data

All manuscripts must include a data availability statement. This statement should provide the following information, where applicable:

- Accession codes, unique identifiers, or web links for publicly available datasets
- A description of any restrictions on data availability
- For clinical datasets or third party data, please ensure that the statement adheres to our policy

Climate data extracted from: Beyer, R. M., Krapp, M. & Manica, A. High-resolution terrestrial climate, bioclimate and vegetation for the last 120,000 years. Scientific Data 7, 236 (2020). https://doi.org:10.1038/s41597-020-0552-1; Yun, K. S. et al. A transient coupled general circulation model (CGCM) simulation of the past 3 million years. Clim. Past 19, 1951-1974 (2023). https://doi.org:10.5194/cp-19-1951-2023; Zeller, E. et al. Human adaptation to diverse biomes over the past 3 million years. Science 380, 604-608 (2023). https://doi.org:10.1126/science.abq1288

Archaeological site data from: ROCEEH Out of Africa Database (ROAD) as published in Kandel, A. W. et al. The ROCEEH Out of Africa Database (ROAD): A large-scale research database serves as an indispensable tool for human evolutionary studies. PLoS One 18, e0289513 (2023). https://doi.org:10.1371/journal.pone.0289513

All archaeological data included in this study is cited and provided within the Supplementary Information. All palaeoclimate data included in this study is cited and provided within the Supplementary Information. Palaeoclimate data is archived and available here: https://figshare.com/s/2c4253aead69d37268a8

## Research involving human participants, their data, or biological material

Policy information about studies with human participants or human data. See also policy information about sex, gender (identity/presentation), and sexual orientation and race, ethnicity and racism.

| | |
|---|---|
| Reporting on sex and gender | No human participants, their data, or biological material were used in this study. |
| Reporting on race, ethnicity, or other socially relevant groupings | No human participants, their data, or biological material were used in this study. |
| Population characteristics | No human participants, their data, or biological material were used in this study. |
| Recruitment | No human participants, their data, or biological material were used in this study. |
| Ethics oversight | No human participants, their data, or biological material were used in this study. |

Note that full information on the approval of the study protocol must also be provided in the manuscript.

# Field-specific reporting

Please select the one below that is the best fit for your research. If you are not sure, read the appropriate sections before making your selection.

☐ Life sciences ☐ Behavioural & social sciences ☒ Ecological, evolutionary & environmental sciences

For a reference copy of the document with all sections, see nature.com/documents/nr-reporting-summary-flat.pdf

# Ecological, evolutionary & environmental sciences study design

All studies must disclose on these points even when the disclosure is negative.

| | |
|---|---|
| Study description | We assembled a comprehensive and curated database of chronometrically dated Pleistocene archaeological sites in Africa and used a species distribution modelling approach to measure changes in the niche breadth of Pleistocene humans in Africa. |
| Research sample | The coordinates and ages of archaeological sites and layers were recorded from: 1) the existing dataset in ROCEEH Out of Africa Database (ROAD), and 2) published literature, as discussed in Methods. |
| Sampling strategy | All published archaeological literature was included whenever possible. To account for sampling bias and differential preservation and fieldwork in the archaeological record, we note that each radiometric date is associated with a specific chronological uncertainty, expressed as a range (most likely date and plus/minus). To take into account such uncertainty, we performed our analyses as follows. We created 100 independent datasets: for each of them, the spatial coordinates of all occurrences (=radiometrically dated archeological layer) were associated to an age that was resampled from the whole chronological range of the date. Such sampling was performed following a truncated normal distribution identified by the mean and the plus/minus as 2-sigma. |
| Data collection | Archaeological sites and deposits were compiled from peer-reviewed journals and books published before May 5, 2021. The Role of Culture in Early Expansions of Humans (ROCEEH) has been integrating archaeological, paleoanthropological, paleontological and paleobotanical information into the ROCEEH Out of Africa Database (ROAD) since 2009. As of May 2021, the team has compiled information on more than 2000 localities and over 12,000 assemblages in Africa and Eurasia dated between three million and 20,000 |

| | |
|---|---|
| | years before present. The information in ROAD is structured into localities (sites) with dated layers that contain assemblages of finds including artifacts, human fossils, paleofauna and plant remains as well as bibliographic sources. For this study, AWK and MW queried ROAD to create a list of archaeological localities in Africa dating between ca. 500,000 and 10,000 years and reviewed the output for accuracy. The output also included the ages of the layers as well as radiometric dating results for calibration. For West Africa, JNC collected additional archaeological site data. For North Africa, EYH collected additional archaeological site data. |
| Timing and spatial scale | Data collection included archaeological site location and dating information for Pleistocene sites in Africa dated to 120,000-14,000 years ago. The ROCEEH Out of Africa Database (ROAD) has been compiling data since 2009. Data collection for this research includes information from peer-reviewed journals and books published before May 5, 2021. |
| Data exclusions | Exclusion criteria were pre-established. Archaeological sites and layers included in this study met the following pre-established criteria: 1) published coordinates, 2) radiometric dates, 3) an age error range less than or equal to 20,000 years, and 4) mean age ≤ 120,000 ≥ 14,000 years. |
| Reproducibility | All attempts to repeat the models were successful and all data and code necessary to reproduce the study are provided. |
| Randomization | We resampled each date 100 times from the truncated normal distribution identified by the mean and the plus/minus as 2-sigma. At the same time, to reduce the conservation bias leading to more recent layers being more frequent, each of those resampled datasets was randomly subsampled to generate a homogeneous distribution in the number of occurrences available through time (thus giving us constant sampling effort through time). For each resampling, we then subset each time slice from the more recent period to the number of occurrences observed in a randomly sampled older slice. To adequately represent the existing climatic space (i.e. background) in our SDMs, each of these resulting datasets was coupled with a random sampling, for each observation, of 200 random locations matched by time. This resulted in n=100 datasets (="repeats") of differently sampled and dated presences and differently sampled background points which we used to repeat our analyses to account for the stochastic sampling performed. |
| Blinding | Blinding was not used during data acquisition or analysis. We assembled a comprehensive database of archaeological site information and do not report experimental results. |

Did the study involve field work? ☐ Yes ☒ No

# Reporting for specific materials, systems and methods

We require information from authors about some types of materials, experimental systems and methods used in many studies. Here, indicate whether each material, system or method listed is relevant to your study. If you are not sure if a list item applies to your research, read the appropriate section before selecting a response.

## Materials & experimental systems

| n/a | Involved in the study |
|---|---|
| ☒ | ☐ Antibodies |
| ☒ | ☐ Eukaryotic cell lines |
| ☐ | ☒ Palaeontology and archaeology |
| ☒ | ☐ Animals and other organisms |
| ☒ | ☐ Clinical data |
| ☒ | ☐ Dual use research of concern |
| ☒ | ☐ Plants |

## Methods

| n/a | Involved in the study |
|---|---|
| ☒ | ☐ ChIP-seq |
| ☒ | ☐ Flow cytometry |
| ☒ | ☐ MRI-based neuroimaging |

# Palaeontology and Archaeology

| | |
|---|---|
| Specimen provenance | No specimens were collected. |
| Specimen deposition | No specimens were collected. |
| Dating methods | No new dates are provided. |

☒ Tick this box to confirm that the raw and calibrated dates are available in the paper or in Supplementary Information.

| | |
|---|---|
| Ethics oversight | No ethical approval or guidance was required as no archaeological materials were collected in this study. |

Note that full information on the approval of the study protocol must also be provided in the manuscript.

# Plants

| | |
|---|---|
| Seed stocks | n/a |
| Novel plant genotypes | n/a |
| Authentication | n/a |
